# Critical insights into the potential risks of antipsychotic drugs to fish, including through effects on behaviour

**DOI:** 10.1111/brv.70031

**Published:** 2025-05-12

**Authors:** Gabrielle Wasser‐Bennett, A. Ross Brown, Samuel K. Maynard, Stewart F. Owen, Charles R. Tyler

**Affiliations:** ^1^ Biosciences, University of Exeter Geoffrey Pope Building, Stocker Road Exeter EX4 4QD Devon UK; ^2^ AstraZeneca, Global Environment Macclesfield Cheshire SK10 2NA UK

**Keywords:** ecotoxicology, neuroactive, pharmaceutical, pollutant, environmental risk assessment, fish, behaviour, fitness

## Abstract

Antipsychotic drugs (APDs) are a diverse class of neuroactive pharmaceuticals increasingly detected in surface and ground waters globally. Some APDs are classified as posing a high environmental risk, due, in part, to their tendency to bioaccumulate in wildlife, including fish. Additional risk drivers for APDs relate to their behavioural effects, potentially impacting fitness outcomes. However, standard ecotoxicological tests used in environmental risk assessment (ERA) do not currently account for these mechanisms. In this review, we critically appraise the environmental risks of APDs to fish. We begin by reading‐across from human and mammalian effects data to standard ecotoxicological effects endpoints in fish. We then explore the wide range of behaviours suitable for ecotoxicological assessment of APDs (and other neuroactive) pharmaceuticals, principally through laboratory studies with zebrafish, and assess the potential for using these behavioural phenotypes to predict adverse individual‐ and population‐level outcomes in wild fish, taking into account phenotypic plasticity. Next, we illustrate the advantages and challenges of measuring and applying behavioural endpoints for fish, including within current regulatory risk assessments. In our final analysis, the implications of relying on apical endpoints for ERA of neuroactive drugs (including APDs) are assessed and recommendations provided for the development of a more refined and tailored mechanistic approach, which would enable more robust assessment of their environmental risk(s).

## I. INTRODUCTION

### (1) Neuroactive drugs and the need for targeted approaches to assess their environmental risk

The increasing use and widespread presence of active pharmaceutical ingredients (APIs) in aquatic environments is recognised as an emerging global threat to wildlife (Arnold *et al*., 2014; Brodin *et al*., 2024). Ecological impacts have been demonstrated for a variety of APIs, with particularly well‐known examples being diclofenac causing declines in vulture populations in Asia (Oaks *et al*., 2004) and ethinyloestradiol inducing feminised fish populations in English rivers (Jobling *et al*., 2006). Dosing of an experimental lake with ethinyloestradiol in Canada induced the collapse of the resident fathead minnow (*Pimephales promelas*) population (Kidd *et al*., 2007). Neuroactive drugs have been highlighted as a potential threat to some forms of aquatic wildlife through alterations to ‘normal’ behaviours (Boxall *et al*., 2012; Arnold *et al*., 2013; Reid *et al*., 2018), notably in fish (Brodin *et al*., 2013; David *et al*., 2018; Cerveny *et al*., 2021; Sumpter & Margiotta‐Casaluci, 2022), however, the significance of these behavioural effects on fish populations is not fully understood. The increasing global detection of antipsychotic drugs (APDs) in surface and ground waters, their continual release [pseudo‐persistence] following manufacture and patient use, as well as features relating directly to the drugs, including their high lipophilicity and tendency for bioaccumulation, evolutionary conservation of their biological targets (amino acid sequences) in vertebrate taxa, and high effectiveness at low doses, makes them a priority freshwater micro‐pollutant, with potential risks to wildlife, including fish (Berninger *et al*., 2016; Escudero *et al*., 2021; Bertram *et al*., 2022).

Traditional apical ecotoxicity endpoints, quantifying survival, growth, sexual development, and reproduction in fish, when the fish are housed under optimal laboratory conditions, may underestimate effects of neuroactive drugs that are capable of disrupting behaviours critical to survival in the wild (e.g. foraging, predation and predator avoidance) and reproduction (e.g. mate selection and courtship). This is of particular concern for fish, which are likely to be exposed to concentrations of increasingly prescribed neuroactive drugs, as they pass through (or indeed, bypass for example *via* combined sewage overflow discharges) municipal wastewater treatment plants into surface water courses (Calisto & Esteves, 2009; Ford & Herrera, 2018; David *et al*., 2018; Saaristo *et al*., 2018). Given the pharmacology of neuroactive drugs and the fact that their target sites are functionally conserved across vertebrate (and invertebrate) phyla, the risks of unintended behavioural effects in a wide range of wildlife seem highly probable (Gunnarsson *et al*., 2008).

Animal behaviours have increasingly been suggested as appropriate endpoints for determining pollutant toxicity due to the sensitivity and integrative nature of the response(s), and their potential for uptake into environmental risk assessment (ERA) is supported by recent technological advances facilitating quantitation of behavioural responses (Hellou, 2010; Ågerstrand *et al*., 2020; Bertram *et al*., 2022, 2025). Nevertheless, behavioural profiling is currently overlooked in ERA (Gunnarson *et al*., 2019; Ford *et al*., 2021) since the regulatory focus is on apical endpoints that could impact a population, such as altered growth, reproduction or survival. Many behavioural endpoints may also have wider applicability beyond studies into the effects of neuroactive drugs, since behavioural alterations (e.g. lethargy, uncoordinated swimming, bottom swimming, surface swimming, cowering/reclusive behaviours) may also result from general narcosis, following disruption of membrane structure and function by other classes of pharmaceuticals and by exogenous substances more generally (van Wezel & Opperhuizen, 1995).

Building the case for behavioural ecotoxicology in the ERA of APDs depends on clearly defining the drug's mode of action to identify relevant assays and differentiate specific effects (e.g. distinct behavioural changes) from general narcotic toxicity, noting that the former is likely to occur at substantially lower chemical exposure concentrations. Given the complex poly‐pharmacology of APDs in humans and lack of mechanistic data for these drugs in fish, we have adopted a broad, integrative approach in this review: identifying patterns in which APD targets are conserved and characterising and quantifying effects on sensory and motor pathways underpinning ecologically relevant behaviours in fish (affecting individual fitness) and phenotypic variation therein. Critically, in these analyses it is important to account for plasticity in behavioural responses among individuals, both within wildlife populations and under different environmental conditions, the latter including changing responses (reaction norms) to environmental stimuli representing opportunities (e.g. prey, potential mates) and threats (e.g. from predators).

### (2) Antipsychotic drugs (APDs) are currently understudied in environmental risk assessment

Since the introduction of APDs in the mid‐20th century, they have become widely prescribed as a primary therapy to provide long‐term intervention for many psychiatric disorders, as well as more recent regulatory approval for off‐target treatments, e.g. dementia, insomnia, and nervous tics (Alexander *et al*., 2011; Hálfdánarson *et al*., 2017). APDs available include older ‘typical’ and newer ‘atypical’ APDs. Both groups predominantly target dopamine receptors, notably the D2 family (D2R), whilst also affecting a range of monoaminergic central nervous system (CNS) receptors (Seeman, 2002). However, atypical APDs have a refined dopamine receptor occupancy that has facilitated more natural neurotransmission, and this, together with dosage reductions, has served to reduce the incidence of neurological side effects (Kapur & Seeman, 2001; Seeman, 2002; Marin & Escobar, 2013).

With the growing burden and debilitating nature of neuropsychiatric disorders in humans, the global APD market is expected to grow to $33.69 billion by 2028, reflecting a compound annual growth rate of medicine usage (3%), market penetration of newer therapies and the COVID‐19 pandemic demand shock (Verdoux, Tournier & Bégaud, 2010; IQVIA Institute Report, 2020; FBI, 2021). A study across 65 countries, found that between 2008 and 2019, APD sales increased at an average rate of ~4% per annum (Brauer *et al*., 2021). In England between 2000 and 2014, APD prescription rates more than doubled, increasing from 0.5% to 1.2% of the population (Stephenson, Karanges & McGregor, 2012; Morrens *et al*., 2015; Shoham *et al*., 2021). This level was closely matched by prescription to those under 18 years old, increasing from 0.057% in 2000 to 0.105% by 2019 (Radojčić *et al*., 2023). The global usage of APDs is also expected to increase as socio‐economic barriers limiting availability in emerging pharmaceutical markets in low‐ and middle‐income countries (LMICs) are lifted [e.g. as patents expire and cheaper generic versions become available (Alabaku *et al*., 2023; Shi *et al*., 2023)].

APDs are generally found in effluent, with especially high prevalence in hospital wastewater (Escudero *et al*., 2021; Helwig *et al*., 2015). As a result of rising prescription volumes (Roberts *et al*., 2018), continual discharge resulting in pseudo‐persistence (Flint *et al*., 2012; Cerveny *et al*., 2021), and a lack of removal *via* conventional wastewater treatment, high‐use APDs are being detected in the environment at concentrations that could affect wildlife. Environmental monitoring data for APDs are mainly concentrated in Europe, North America and some parts of Asia – thus largely excluding countries where sewage goes untreated, elevating risk further (Wilkinson *et al*., 2022). For example, the removal efficiency for aripiprazole in a relatively sophisticated American wastewater treatment plant (WwTP) has been reported at 68%, but there is a very high likelihood for much lower removal efficiencies in less‐advanced systems, which exist notably in LMICs (Calisto & Esteves, 2009; Verlicchi, Aukidy & Zambello, 2012; Fekadu *et al*., 2019; Subedi & Kannan, 2015). Additionally, APD prescription data are largely absent from emerging markets in LMICs. Based on limited data it is estimated that APD concentrations in wastewater discharges in LMICs may be up to 1000× higher than those found in Europe (Fekadu *et al*., 2019). It should also be recognised, however, that there is great uncertainty about the contribution of drugs in surface water discharged in western global regions through combined sewage overflows (CSOs), which do not receive any treatment. This input may not be insignificant, as, for example, in 2023 there were 3.6 million hours of these CSOs into UK rivers (EA, 2024).

APDs (Table 1) are increasingly being recognised for their ecotoxicity. Chlorpromazine, for example, features in the top 15 ‘most hazardous compounds in hospital effluent’ and is generally classified as ‘high concern’ to aquatic organisms (Lacoursiere & Spohn, 1976; Fick *et al*., 2010; Orias & Perrodin, 2014; Sangion & Gramatica, 2016; Golbaz *et al*., 2021). Similarly, haloperidol, which has held World Health Organisation (WHO) essential medicine status since 1977, often ranks highly in assessments of the relative risks of drugs to the environment (Roos *et al*., 2012; Grabicová *et al*., 2017; Malev *et al*., 2020; Cerveny *et al*., 2021; WHO, 2021). Another legacy compound, Methotrimeprazine, was recently prioritised due to its ‘high‐risk, data‐poor’ categorisation (Cannata *et al*., 2023). The more recently registered compounds aripiprazole and brexpiprazole have raised Persistence, Bioaccumulation and Toxicity (PBT) concerns, yet have largely escaped environmental monitoring scrutiny (Howard & Muir, 2010; EMA, 2018; Wronski & Brooks, 2023).

**Table 1:** Measured environmental concentrations, physico‐chemical properties and preliminary predicted no‐effect data. The pharmaceutical class of antipsychotics [Anatomical Therapeutic Chemical (ATC) code: cluster N05A] is comprised of 45 active pharmaceutical ingredients (APIs) (all included). Antipsychotic drugs (APDs) are grouped by generation (Gen 1–3) and include their CAS number. Maximum measured environmental concentration (MEC), influent and/or effluent and surface water, are from Wronski & Brooks (2023). Critical Environmental Concentrations (CECs) – the surface water concentration expected to cause a pharmacological effect in fish – were retrieved from Fick *et al.* (2010) using the EPI Suite™ KowWin program; predictions are based on neutral forms of the API. CEC(*) takes into account ionisation to obtain a value [calculated using an equation derived from Fitzsimmons *et al.* (2001) for pH‐corrected octanol–water partition coefficient (*D*_ow_); log*D*_ow_ 1–8] that is comparable with the predicted no‐effect concentration (PNEC). PNECs were retrieved from EMA EPAR assessment reports (see EMA, 2014, 2016, 2018, 2021), if available, for compounds that were released after 2006, unless otherwise stated. Human therapeutic plasma concentrations (HTPCs) are retrieved from Schulz *et al.* (2020) using the lowest therapeutic normal concentration. Bioconcentration Factor (BCF) and Log*D* @ ph 7.4 (partition coefficients, i.e. measures of lipophilicity) were predicted using ChemSpider (http://www.chemspider.com). Note that the environmental effects (ecotoxicity) of the majority of these APIs have yet to be investigated. Legacy [Gen 1] compounds particularly lack measured ecotoxicity data. Note also that some compounds have been discontinued from use. Consistency and reliability of predicted ecotoxicity data also remains in question. AF, assessment factor; LOQ, limit of quantification; ND, not detected; NOEC, no observed effect concentration; n.s, not stated; QSAR, quantitative structure–activity relationship.

API (CAS number) [Gen]	Maximum MEC influent/effluent concentration (ng/l)	Maximum MEC surface water (ng/l)	CEC (ng/l) un‐ionised	CEC ^(*)^ (ng/l) ionised	PNEC (units) [Most sensitive species or QSAR/ECOSAR]	HTPC mg/l (ng/l)	BCF	LogD
**Chlorpromazine** (50–53‐3) [1]	520	41	36	587.8	0.088^1^ ng/l [*Daphnia*]	0.03 (30,000)	80.07	3.42
**Haloperidol** (52–86‐8) [1]	2730	100	6.5	68.5	1400^2^ ng/l [*Zebrafish*]	0.001 (1000)	38.78	2.65
**Promazine** (58–40‐2) [1]	–	–	–	642.8	3740^3^ (ng/l) [QSAR]	0.01 (10,000)	21.87	2.69
**Fluphenazine** (69–23‐8) [1]	7.4	–	–	6.1	6670^3^ (ng/l) [QSAR]	0.001 (1000)	654.58	4.12
**Trifluoperazine** (117–89‐5) [1]	ND	ND	–	4.2	3440^3^ (ng/l) [QSAR]	0.001 (1000)	835.29	4.34
**Perazine** (84–97‐9) [1]	–	–	–	158.0	3140^3^ (ng/l) [QSAR]	0.01 (10,000)	208.03	3.55
**Prochlorperazine** (58–38‐8) [1]	ND	ND	121	5.2	2490^3^ (ng/l) [QSAR]	0.001 (1000)	684.82	4.22
**Prothipendyl** (303–69‐5) [1]	–	–	–	8271.5	8280^3^ (ng/l) [QSAR]	0.03 (30,000)	3.34	1.70
**Benperidol** (2062‐84‐2) [1]	–	–	–	41.0	4840^3^ (ng/l) [QSAR]	0.001 (1000)	60.06	2.97
**Droperidol** (548–73‐2) [1]	–	–	1130	–	22060^3^ (ng/l) [QSAR]	–	54.04	2.78
**Fluspirilene** (1841‐19‐6) [1]	–	–	–	3.0	9980^3^ (ng/l) [QSAR]	0.0001 (100)	56.10	3.16
**Penfluridol** (26864–56‐2) [1]	–	–	–	3.6	2680^3^ (ng/l) [QSAR]	0.004 (4000)	3592.43	5.27
**Zuclopenthixol** (53772–83‐1) [1]	<LOQ	–	–	22.5	4030^3^ (ng/l) [QSAR]	0.004 (4000)	759.94	4.17
**Perphenazine** (58–39‐9) [1]	45	0.34	9.9	5294.2	0.71^1^ ng/l [*Daphnia*]	0.6 (600,000)	446.51	3.90
**Pimozide** (2062‐78‐4) [1]	–	–	–	6.2	7620^3^ (ng/l) [QSAR]	0.003 (3000)	1153.98	4.77
**Pipamperone** (1893‐33‐0) [1]	2430000	961.3	n/a	30905.9	36600^3^ (ng/l) [QSAR]	0.1 (100,000)	6.18	1.61
**Chlorprothixene** (113–59‐7) [1]	2260	51	27	198.3	990^3^ (ng/l) [QSAR]	0.02 (20,000)	194.09	3.83
**Clopenthixol** (982–24‐1) [1]	–	–	–	12.6	4030^3^ (ng/l) [QSAR]	0.002 (2000)	652.19	4.10
**Flupentixol** (2709‐56‐0) [1]	19	370	3.9	2.0	3120^3^ (ng/l) [QSAR]	0.0005 (500)	1061.23	4.36
**Pipotiazine** (39860–99‐6) [1]	–	–	–	78.9	1120^3^ (ng/l) [QSAR]	0.001 (1000)	19.45	2.56
**Thioridazine** (50–52‐2) [1]	ND	ND	16	1252.0	220^3^ (ng/l) [QSAR]	0.1 (100,000)	99.56	3.69
**Periciazine** (2622‐26‐6) [1]	–	–	–	440.3	6710^3^ (ng/l) [QSAR]	0.005 (5000)	22.81	2.49
**Cyamemazine** (3546‐03‐0) [1]	ND	–	–	56.0	2430^3^ (ng/l) [QSAR]	0.0009 (900)	23.86	2.71
**Methotrimeprazine** (60–99‐1) [1]	–	–	–	188.9	2750^3^ (ng/l) [QSAR]	0.005 (5000)	40.84	3.02
**Levosulpiride** (23672–07‐3) [1]	–	–	–	–	50600^3^ (ng/l) [QSAR]	–	1.00	−0.99
**Bromperidol [1]** (10457–90‐6)	–	–	–	809.0	3800^3^ (ng/l) [QSAR]	0.012 (12,000)	39.35	2.66
**Melperone** (3575‐80‐2) [2]	73	–	2384	8706.6	7800^3^ (ng/l) [QSAR]	0.03 (30,000)	3.73	1.66
**Sertindole** (106516–24‐9) [2]	–	–	–	858.1	12300^3^ (ng/l) [QSAR]	0.05 (50,000)	139.95	3.50
**Clothiapine** (2058‐52‐8)[2]	–	–	–	–	2970^3^ (ng/l) [QSAR]	–	193.27	3.36
**Clozapine** (5786‐21‐0) [2]	14,430	78,300	321428	21448.5	3130^4^ (ng/l) [*Algae*]	0.35 (350,000)	54.85	2.72
**Loxapine** (1977‐10‐2) [2]	–	–	–	218.6	0.00528^3^ (ng/l) [QSAR]	0.005 (50,00)	90.26	2.93
**Risperidone** (106266–06‐2) [2]	4286.7	3683.3	129	4770.0	5800^2^ (ng/l) [*Bluegill sunfish*]	0.02 (20,000)	9.06	1.81
**Paliperidone** (144598–75‐4) [2]	12.8	0.6	–	12967.7	250,000 (ng/l) [*Daphnia*]	0.02 (20,000)	1.75	0.88
**Ziprasidone** (146939–27‐7) [2]	4	–	356	405.0	110^1^ (ng/l) [*Daphnia*]	0.02 (20,000)	155.36	3.4
**Zotepine** (26615–21‐4) [2]	–	–	–	66.4	1890^3^ (ng/l) [QSAR]	0.01 (10,000)	350.57	4.07
**Asenapine** (65576–45‐6) [2]	–	–	–	235.3	12.5^8^ μg/kg ww [*Fish*]	0.001 (1000)	4.52	1.82
**Amisulpride** (71675–85‐9)[2]	1930	460	–	108972.3	5400^5^ (ng/l) [ECOSAR]	0.1 (100,000)	1.00	−0.43
**Tiapride** (51012–32‐9) [2]	ND	ND	–	1154086.4	8720^6^ (ng/l) [n.s]	1 (1,000,000)	1.00	−1.07
**Sulpiride** (15676–16‐1) [2]	15,359	13418	138,347	1149031.2	8^1^ (ng/l) [*Algae*]	1 (1,000,000)	1.00	−0.99
**Lurasidone** (367514–87‐2) [2]	–	–	–	96.4	2600 (ng/l) [*Daphnia*]	0.015 (15,000)	503.71	4.09
**Olanzapine** (132539–06‐1) [2]	520	54274	3634	4219.0	1100^2^ (ng/l) [*Fathead minnow*]	0.02 (20,000)	11.12	1.9
**Quetiapine** (111974–69‐7) [2]	24,000	346.7	290,938	11971.6	10,000^7^ (ng/l) [*Fathead minnow*]	0.1 (100,000)	30.52	2.29
**Aripiprazole** (129722–12‐9) [3]	150	8.3	1024	142.1	261 (ng/l) [*Daphnia*]	0.1 (100,000)	3248.12	4.99
**Brexipiprazole** (913611–97‐9) [3]	–	–	–	–	5,600 (ng/l) [*fishNOEC*·^*AF=10*^]	0.04 (40,000)	–	–
**Cariprazine** (839712–12‐8) [3]	–	–	–	27.8	7540^3^ (ng/l) [QSAR] 2023	0.01 (10,000)	1554.89	4.59

^1^ Orias & Perrodin (2014); ^2^Swedish National Formulary of Drugs (FASS) (accessible at www.fass.se/LIF/startpage, see *FASS* link in references for *Haldol®, Olanzapine & Risperdal®*, note that results vary depending on approaches to prevalence and forecasting data); ^3^QSAR, 2023 taken from Cannata *et al*. (2023); ^4^Minguez *et al*. (2016); ^5^Helwig *et al*. (2015); ^6^Gosset *et al*. (2021); ^7^AstraZeneca (2023); ^8^NORMAN Ecotoxicity Database (http://www.norman‐network.com/nds/ecotox/) provides QSAR‐based predicted values for biota, the fish value is provided here in the units (μg/kg ww) provided by NORMAN.

### (3) Target conservation and mode of action of APDs in fish (with a focus on zebrafish)

**Fig. 1:**
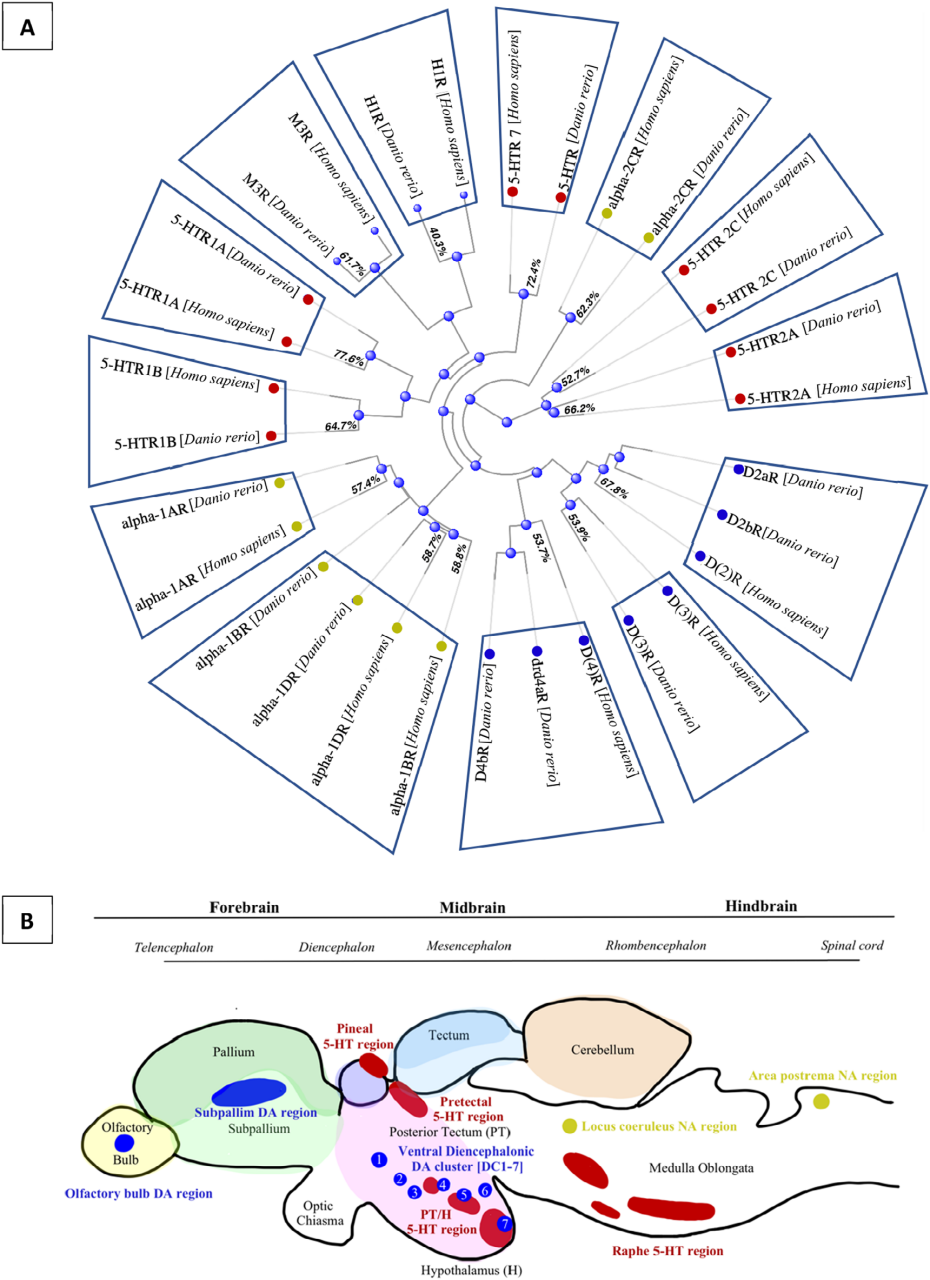
Primary drug targets of antipsychotic compounds. (A) Protein sequences obtained from NCBI HomoloGene Downloader and distance tree of results produced using BLAST pairwise alignment. Species level conservation (%) by majority vote from ECOdrug (https://ecodrug.org/). (B) Sagittal zebrafish brain schematic adapted from (Gaspar & Lillesaar, 2012), approximately identifying the most significant populations of primary pharmacological targets of APDs: dopaminergic (DA, in blue), serotonergic (5‐HT, in red) and noradrenergic (NA, in yellow). Significant brain regions are highlighted for context (Chia *et al*., 2022; Rubenstein & Rakic, 2013; Matsui & Sugie, 2017; Godoy *et al*., 2020).

Drug target conservation is a major factor determining the likelihood that a drug will induce an effect in a non‐target organism. The zebrafish (*Danio rerio*) genome (current genome assembly: GRCz11; http://www.ensembl.org/Danio_rerio/Info/Index) has 86% orthology with humans for over 1300 genes constituting medicinal drug targets (Gunnarsson *et al*., 2008; Verbruggen *et al*., 2018) (see Fig. 1A), indicating physiological susceptibility to human pharmaceuticals (Guo, 2004; Owen *et al*., 2007; Gunnarsson *et al*., 2008; Howe *et al*., 2013; Furuhagen *et al*., 2014).

The zebrafish is widely used to model complex drug‐induced phenotypes in pharmaceutical development and human safety assessment, as well as in environmental risk assessment (Gerlai, 2003; Sumanas & Lin, 2004; Rihel *et al*., 2010; Kawahara *et al*., 2011; Khan *et al*., 2017; Audira *et al*., 2020). Zebrafish are particularly amenable to laboratory effects testing due to their small size, high reproductive rate, short generation time (reaching adulthood within 3 months), and relatively low maintenance costs, and their transparent embryo–larval stages enable the visualisation of neurodevelopment (Sumanas & Lin, 2004; Mrinalini, Kumar & Manasa, 2023). Transgenic zebrafish with pan‐neuronal or selected neuronal fluorescence markers in mutant lines that lack skin pigmentation are also widely available and particularly valuable for neurological profiling.

Furthermore, the presence of major drug targets for APDs (dopamine and serotonin receptors) in zebrafish (Verbruggen *et al*., 2018; see Fig. 1B) has led to a surge in their use for neurochemical and behavioural studies, and especially the study of neuroactive contaminants (Guo, 2004; Barros *et al*., 2008; Flinn *et al*., 2008; Makhija & Jagtap, 2014). As such, standardised behavioural screening with zebrafish could offer a powerful tool to help determine environmental consequences of high‐priority neuroactive drugs (Rihel & Schier, 2012).

The mode of action (MoA) of APDs in fish remains uncertain, though drug action is largely considered to centre around the monoaminergic system, potentially driving responses at levels below general toxicity. Thus, it is possible to differentiate between narcosis and a more clearly defined MoA‐related neurological effect.

In zebrafish, the dopaminergic (DA) neurons are concentrated in the basal forebrain and diencephalon, with specialised dopamine neurons found locally within the sensory system (retina and olfactory bulb) (Fig. 1B). Zebrafish lack dopaminergic populations in the midbrain and hindbrain, but the forebrain neuronal dopamine system is thought to perform the function of midbrain dopaminergic neurons in humans (Rink & Wullimann, 2001; Wasel & Freeman, 2020). Dopamine cell bodies are abundant from an early age in zebrafish, with dopaminergic neurons present as early as 18–19 h post fertilisation (hpf) (Panula *et al*., 2010). Although teleost fish, including the zebrafish, possess three more dopamine receptor types than found in mammals, mammalian D1‐like and D2‐like dopamine receptors are present, with functional similarity to mammalian brains (Winberg & Nilsson, 1993; Van der Ven *et al*., 2006; Panula *et al*., 2010). Similarly, dopamine plays a key role in the physiological homeostasis and behavioural regulation of fish, including critical behaviours such as movement (Lambert, Bonkowsky & Masino, 2012), and those involved in reproduction (Dufour *et al*., 2010). A balance between the dopamine and serotonin system is equally important for the regulation of normal neurological and behavioural functionality, such as sociality (Teles *et al*., 2013).

The organisation of the serotoninergic (5‐HT) system throughout the CNS in zebrafish (and other fish species) closely resembles that found in mammals, including humans. The zebrafish possesses three serotonin receptor subtypes compared to five in humans (Panula *et al*., 2010). Nevertheless, 5‐HT2R (notably antagonised by APDs) is present in both, and receptor expression has been noted throughout the brain (Fig. 1B), as well as in peripheral tissues during early zebrafish development (from 2 days post fertilisation, dpf) (Bortolato, Chen & Shih, 2010; Lillesaar, 2011; Prieto *et al*., 2012; Schneider *et al*., 2012; Prasad, Ogawa & Parhar, 2015a,b). The role of serotonin in mediating physiological state (e.g. muscle function and blood pressure) (Connors, 2013), as well as mood and behaviour (e.g. locomotion, appetite, cognition, and reproduction and social behaviours) is clearly evident in both humans (Winberg & Nilsson, 1993; Saxena, 1995; Kema, De Vries & Muskiet, 2000; Marin & Escobar, 2013) and fish (Lucki, 1998; Lillesaar., 2011; Prasad *et al*., 2015b; David *et al*., 2018). Serotonergic involvement in predator avoidance (McLean & Fetcho, 2004), locomotion and social behaviour (aggression, social cohesion, reproduction), are especially well demonstrated in fish (Winberg & Nilsson, 1993).

APDs also target adrenoreceptors (α1R and α2R) within the noradrenaline (NA) system, with the aim of improving cognition, motivation, and arousal and reducing anxiety in patients (Kim *et al*., 2002; Marino *et al*., 2005; Bauknecht & Jékely, 2017), although there is a lack of direct clinical evidence for this (Maletic *et al*., 2017). Noradrenaline is an evolutionarily ancient and conserved neurotransmitter involved in muscle contraction, insulin release, cardiac output, stress response (fight or flight), memory and arousal in humans (Glavin, 1985; Tully & Bolshakov, 2010, O'Donnell *et al*., 2012). Activation of α1R and α2R‐like adrenoreceptors in the locus coeruleus (Fig. 1B) and peripheral organs of zebrafish forebrain (resembling humans) has likewise been shown to mediate arousal (Singh, Oikonomou & Prober, 2015) and locomotion, as well as pigmentation (Ruuskanen *et al*., 2005).

Considering the substantial role of monoaminergic neural systems in regulating key behaviours, even minor modifications may induce a substantial fitness‐altering shift, both from direct antagonism/agonism or exposure altering neuronal morphology, leading to altered neuro‐expression, inducing shifts in physiology and behaviour. This information enables prediction of suitable endpoints to determine likely effects in non‐target organisms (Bound & Voulvoulis, 2004; Ankley *et al*., 2010; Saussereau *et al*., 2013; David *et al*., 2018).

Antipsychotic therapies also often interact with histaminergic (H1), and muscarinic (M1 and M3) receptors. Frequently, they are associated with inducing a diverse range of metabolic effects (cardiovascular effects, hyperlipidemia, insulin resistance and obesity) in patients (Miyamoto *et al*., 2004; He, Deng & Huang, 2013; Siafis *et al*., 2018); thought to be mediated through insulin secretion and heightened prolactin, as well as behaviours such as increased appetite, and reduced activity and motivation (Kroeze *et al*., 2003; Vehof *et al*., 2011; Siafis *et al*., 2018). The muscarinic system in zebrafish parallels that occurring in mammals, retaining (but duplicating) all *CHRM1‐5* genes which encode for muscarinic receptors (Pedersen, Bergqvist & Larhammar, 2018), and has been linked to changes in higher‐level cognition (Kim *et al*., 2010; Cognato *et al*., 2012), and locomotion (Siregar *et al*., 2021). The histaminergic system is also well conserved in zebrafish (Kaslin & Panula, 2001) and receptor antagonism has been linked to changes in zebrafish swimming, exploration, anxiety, and cognitive capacity (Peitsaro *et al*., 2004, 2007).

Whilst secondary (off‐target) effects are rationalisable, primary pharmacological effects, related to the above primary drug targets, are likely to occur at substantially lower exposure concentrations than apical toxicity endpoints and are the main focus of this review. Comparable environmental concentrations are provided alongside reviewed experimental exposure and effects data to support interpretation of results. It is noteworthy that environmentally relevant dose–response relationships have rarely been examined in mainstream research within behavioural toxicology (see Margiotta‐Casaluci *et al*., 2014).

## II. ENVIRONMENTAL LEVELS OF APDs AND POTENTIAL RISKS POSED TO FISH

Concentrations of APDs measured in the environment (in wastewater effluent and surface waters) are found at low‐levels: parts‐per‐trillion (ng/l) to parts‐per‐billion (μg/l) (Roberts *et al*., 2018; Perez *et al*., 2022). Measured concentrations of APDs in receiving aquatic environments (Subedi & Kannan, 2015; Wronski & Brooks, 2023) often exceed: (*i*) the action limit of 0.01 μg/l triggering ecotoxicity testing (EMA, 2024); (*ii*) Critical Environmental Concentrations (CECs) equating to pharmacologically active concentrations in fish (Huggett *et al*., 2003; Fick *et al*., 2010; Gunnarsson *et al*., 2019), and in some cases (*iii*) Predicted No Effect Concentrations (PNECs) derived from apical ecotoxicity tests (Table 1).

APDs are present in the environment largely due to untreated (~ 48% of wastewater produced globally is released untreated) and variable, including low, removal efficiencies for APDs and their metabolites in WwTPs (Calisto & Esteves, 2009; Verlicchi *et al*., 2012; Jones *et al*., 2020). Some APDs are excreted predominately as the parent compound (e.g. paliperidone ~60%), whereas others are extensively metabolised, with only a small fraction of parent APDs (e.g. aripiprazole 18%) being excreted in urine and faeces (Calisto & Esteves, 2009; Sheehan *et al*., 2010; Subedi & Kannan, 2015). Several APD metabolites retain physiological activity, and in some cases exhibit equivalent potency to the parent compound (Spina & De Leon, 2006). Additionally, during wastewater treatment, certain APD metabolites can be converted back into their parent compounds, for example through processes such as deconjugation (Daughton, 2014; Argaluza *et al*., 2021). This phenomenon has been documented for risperidone (Fick *et al*., 2011), highlighting the complexities of APD metabolism in environmental contexts. Many APDs are also resistant to environmental (biotic and abiotic) degradation, such as both chlorpromazine and aripiprazole that are resistant to photolytic hydrolysis (Srinivas *et al*., 2008; Trawiński & Skibiński, 2016; Escudero *et al*., 2021). In cases where APDs are more readily degraded, toxic and persistent products can be produced, e.g. abiotic photoproducts of chlorpromazine (Trautwein & Kümmerer, 2012; Pereira *et al*., 2018). APDs are also generally lipophilic with a tendency to bioaccumulate in lipid‐rich tissues, crucially in the brain of fish, affecting neurotransmission and behaviour (Nallani *et al*., 2016; Grabicová *et al*., 2017; David *et al*., 2018; Malev *et al*., 2020; Cerveny *et al*., 2021). Furthermore, even when not detected within estuarine water samples, the APD risperidone has still been detected at high frequency (65–72%) in brain, muscle, and liver tissues, at concentrations ranging up to 0.6 ng/g, in multiple fish species (Duarte *et al*., 2023).

Indeed, due to their aforementioned persistence, bioaccumulation potential and physiological potency, APDs are overrepresented in high‐hazard categories and are, in fact, considered one of the most hazardous API compound classes, and in some cases have been shown to be toxic to fish (Sanderson *et al*., 2004, Ramström *et al*., 2020). Screening of over 1000 pharmaceuticals has established that out of the 35 pharmaceuticals listed as potentially environmentally toxic and requiring prioritisation or greater experimental validation, six are APDs (chlorpromazine, chlorprothixene, triflupromazine, fluspirilene, pimozide, and aripiprazole) (Sangion & Gramatica, 2016). It is noteworthy that even when effects data used in the above prioritisation were lacking (e.g. lack of a clearly defined PNEC for the first‐generation APD chlorpromazine), derived Risk Quotients [RQ = Measured Environmental Concentration (MEC) / PNEC] were clearly above the threshold value of 1 (Table 1, e.g. for chlorpromazine RQ = MEC_(max surface water)_/PNEC = 41/0.088 = 466). For newer, third‐generation APDs with complete ERAs, e.g. aripiprazole (EMA, 2016), the RQ calculated using the EMA's PEC_(surface water)_ of 251 ng/l also approaches the threshold (0.962 for aripiprazole), indicating a narrow margin of safety.

Furthermore, MECs for clozapine, risperidone and chlorpromazine have been recorded in treated municipal effluent and hospital waste streams at concentrations 600× higher than safe exposure levels based on effects on apical endpoints (i.e. PNECs for growth, development, and reproduction) (Reichert, Souza & Martins, 2019). The application of a dilution factor (DF), as per Keller *et al*. (2014) [25th percentile (DF = 318), derived for data from Reichert *et al*. (2019) in Brazil], to these three high‐RQ examples (clozapine, risperidone and chlorpromazine) still results in an RQ >1 (1.26–2, 2.9–3.1 and 18.6 respectively). Note that in some cases, higher MECs have been reported for all three compounds, as well as much lower dilution factors (e.g. a median tenfold lower in the UK), which would ultimately lead to even higher RQs.

Despite this, ERAs are lacking for APDs that received marketing authorisation prior to 2006, the year in which EU Guidelines for the ERA of human pharmaceuticals were first published (EMA, 2006, updated 2024). In fact, as a distinct group of APIs, APDs are among those that most lack ERA data, with 30% lacking a chronic PNEC value (Orias & Perrodin, 2014).

Application of cross‐species extrapolation (‘read‐across’) approaches for assessing risks of APDs to fish has been proposed based on limited data from other neuroactive drug classes (Margiotta‐Casaluci *et al*., 2014). Considering target conservation and using available human therapeutic plasma concentration (HTPC) together with the predicted blood plasma concentration in fish, the ‘Fish Plasma Model’ (FPM) proposed by Huggett *et al*. (2003), should enable a reasonable estimation of risk to fish (Huggett *et al*., 2003; Fick *et al*., 2010; Rand‐Weaver *et al*., 2013). However, David *et al*. (2018) argued that read‐across does not work from humans to fish for neuroactive substances, including APDs, as they have a tendency to bioaccumulate in the lipid‐rich brain, reaching concentrations many times higher than that found in the blood plasma. Other recent work has also shown that blood:water partitioning of pharmaceuticals may not be well predicted by the FPM, based on hydrophobicity and passive uptake across fish gills (Escher *et al*., 2020) and that the fraction of unbound API in blood plasma (available for pharmacological action) may differ considerably in fish compared to humans (Henneberger *et al*., 2020).

Furthermore, despite some functional similarities between human and fish metabolism, differences in phase 1 enzymes [e.g. cytochrome P450 (CYP) enzymes] are likely to affect the efficacy of pharmaceutical clearance and drug breakdown, and affect their accumulation in body tissues, ultimately affecting their toxicity (Buhler & Wang‐Buhler, 1998; Gomez, Constantine & Huggett, 2010; Smith *et al*., 2010; Matthee *et al*., 2023). The effects of APDs on metabolism and their propensity to be metabolised remain largely unstudied and extrapolation from humans to fish is not reliable, as demonstrated for other neuroactives (Heel *et al*., 1981; Nakamura *et al*., 2008; Chen *et al*., 2017; Cerveny *et al*., 2021). In one notable case, the APD olanzapine was found to affect both lipid metabolism and gut microbiome in the common carp (*Cyprinus carpio* L.), driving lipid accumulation in the liver (Chang *et al*., 2022). Recently, APDs (quetiapine and clozapine) were also found to have a weakly inhibitory effect on CYP homolog activity in rainbow trout (*Oncorhynchus mykiss*) *in vitro*, although this was not found universally for all APDs tested, nor were these effects examined at environmentally relevant levels (Pihlaja *et al*., 2024).

## III. FISH BEHAVIOURAL ECOTOXICOLOGY AND APPLICATIONS FOR STUDIES ON APDs

### (1) Recent developments

Quantifying changes in critical (survival, growth, reproduction) behaviours of ecological significance (Wong & Candolin, 2015) is acknowledged as an effective risk assessment approach due to significantly (10–1000×) greater sensitivity to chemical exposure compared to traditional apical endpoints of survival, growth, and reproductive output (Little & Finger, 1990; Robinson, 2009; Melvin & Wilson, 2013). Recent studies have applied a range of behavioural methodologies to assess the effects of APDs in fish at multiple life stages and to assess impacts on individual fish, their offspring and their populations (see online Supporting Information, Table S1). These may not have been identified by traditional endpoints.

Currently, the greatest barriers to the integration of behavioural endpoints in chemical risk assessment are the perceived shortfalls in consistency, precision and accuracy of non‐standardised endpoints, as well as uncertainty in extrapolation of individual laboratory studies to environmentally relevant population‐level effects (Ågerstrand *et al*., 2020; Ford *et al*., 2021). While it is often argued that apical population‐level effect measures integrate behavioural and physiological effects, it is generally the case that behavioural effects precede apical effects and that they underpin fitness in terms of survival (e.g. predation and predator avoidance behaviours) and reproduction (e.g. mating behaviours) in the wild. Moreover, for APDs, appreciating that behavioural modification is a consequence of their multi‐target MoA can help refine current guidelines and increase confidence in the ERA of these compounds. However, a major challenge in linking behavioural responses to relevant fitness outcomes lies in defining the thresholds for behavioural change that meaningfully impact fitness, survivorship, etc. Additionally, building confidence in the use of behavioural measures for assessing APDs also requires a deeper understanding of the phenotypic plasticity in behavioural responses to these neuroactive drugs and this has received little attention to date.

**Fig. 2:**
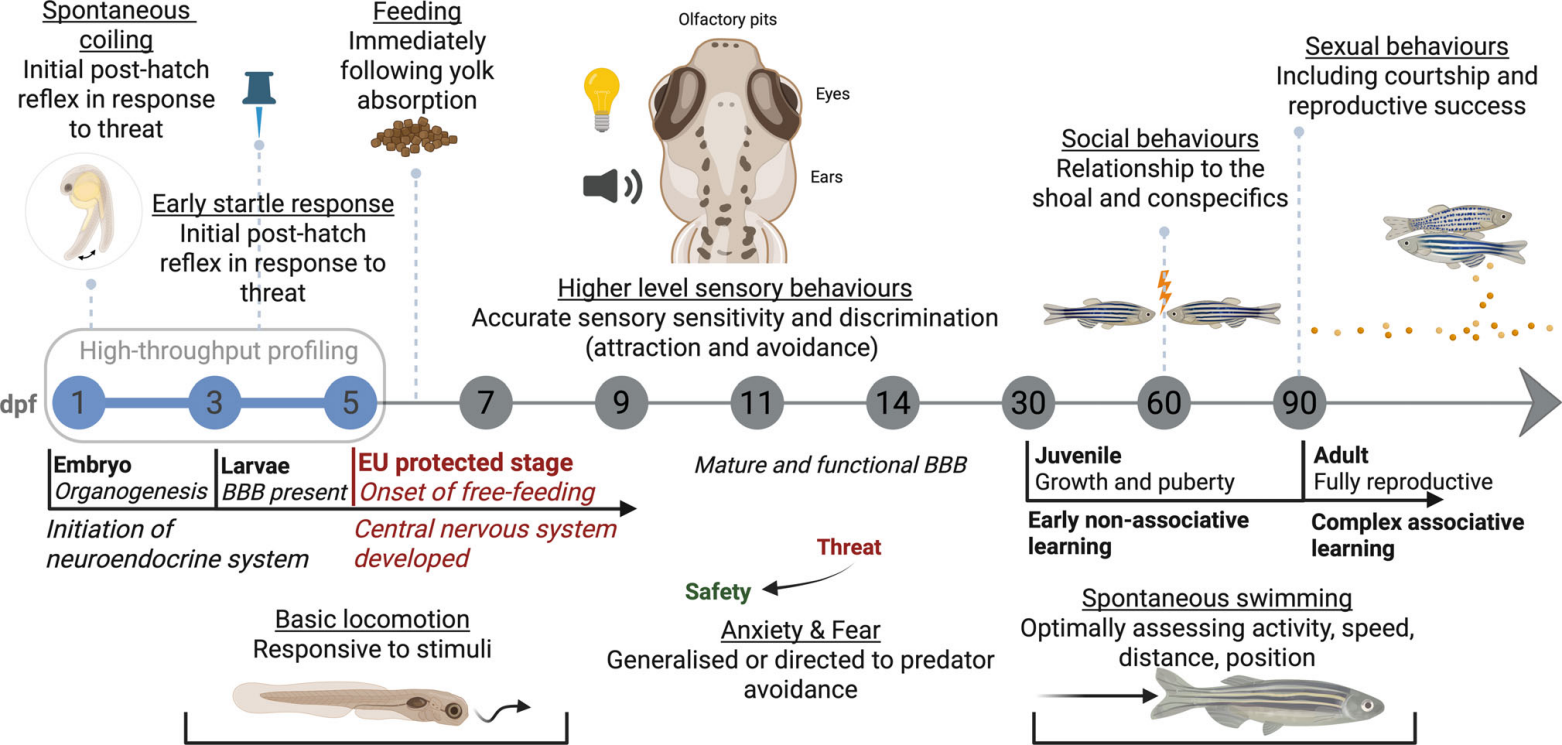
Behaviours of zebrafish across its life cycle with potential for use in environmental risk assessments. Behavioural phenotypes recapitulate the ongoing state of the central nervous system (CNS), from the neural networks to sensory input, processing and ultimately translation to a variety of defined (innate and learned) behavioural outputs. Age of the fish is indicated by the central horizontal line (dpf = days‐post‐fertilisation). Major developmental stages are shown against the key behavioural features that can be quantified. The behavioural repertoire will improve over time (e.g. feeding begins following yolk absorption, this behaviour becomes refined with the development of greater locomotor capabilities and jaw/gape size increase). BBB, blood–brain barrier. EU protected stage, refers to the onset of free feeding (5 dpf) in zebrafish, at which point they are considered to be experimental animals protected under EU regulation (EU Directive 2010/63). Created in BioRender.

There is, however, growing recognition of the merits of tailoring behavioural studies to pharmaceutical mode of action (for APDs, largely focused on the stimulation–perception–locomotion axis, with multiple examples discussed below) and animal developmental stage (Fig. 2) in the ERA of pharmaceuticals (Daughton & Ternes, 1999; De Esch *et al*., 2012; Legradi *et al*., 2018), while also reducing and refining animal testing in research (Message & Greenhough, 2019).

In addition to the zebrafish, several other common laboratory fish models are now routinely applied in behavioural ecotoxicology, the most common of which include medaka (*Oryzias latipes*), fathead minnow and three‐spined stickleback (*Gasterosteus aculeatus*), all recommended Organisation for Economic Co‐operation and Development (OECD) freshwater test species (Norrgren, 2012; Steele, Mole & Brooks, 2018). The three‐spined stickleback, in particular, has a long history as a model for behavioural studies and is best known for its characteristic courtship and aggression responses, a key target for APD MoA (Norton & Gutiérrez, 2019), which have greatly facilitated effects analysis of chemical pollutants on stickleback reproductive behaviours (Bell, 2001). However, the vast majority of behavioural studies assessing APDs (>75%) to date have been conducted in zebrafish (see Table S1). Various saltwater fish, including anemonefish (e.g. *Amphiprion ocellaris* and *A. clarkii*), and marine medaka (*O. melastigma*) are also being used in ecotoxicological testing for marine ERAs (Dong *et al*., 2014; Pouil, Besson & Metian, 2022), but to date there has been a lack of studies assessing behavioural effects of APDs in marine fish (see Table S1). While data on the occurrence of APDs in marine systems is limited, available MECs generally fall within the range observed in freshwater environments (Wronski & Brooks, 2023).

Recent efforts have been made to increase the complexity and environmental relevance of behavioural ecotoxicology, with ongoing *in‐situ* studies intended both to validate laboratory‐to‐field read across, as well as potentially to assess better the real‐world risk to fish. Currently, field assessments of APDs in fish focus largely on tissue accumulation (see Table S1), however, more broadly, effects of neuroactive pharmaceuticals on wild fish behaviours – such as activity, foraging, boldness, and sociality – have been demonstrated, showing good comparability with laboratory‐exposure studies (Brodin *et al*., 2013). These effects have also been linked to broader ecological changes in terms of environmental resource use, including more bold use of space (Klaminder *et al*., 2016). Such increased boldness may be ecologically advantageous in terms of increased foraging and food intake, but disadvantageous in terms of increased risk of predation. Boldness (risk‐taking) is a key trait that could be influenced by APDs due to its association with dopamine, specifically the D2 receptor. Boldness is often explored through space use as part of the novel tank test, light/dark or predator test (Thörnqvist *et al*., 2018).

The evidence for such effects is mixed across different fish species, highlighting the need to consider differential species sensitivity and plasticity to behavioural effects. For example, no evidence of increased predation has been found in ‘bolder’ wild perch (*Perca fluviatilis*) populations exposed to the anxiolytic drug oxazepam (Klaminder *et al*., 2016; Lagesson *et al*., 2018; Fahlman *et al*., 2021), whereas exposure to the same drug was linked to increased predation in a study on salmon (*Salmo salar*) in a natural lake ecosystem (Klaminder *et al*., 2019). No comparable evidence currently exists for APD exposure to fish in natural systems. Nevertheless, behavioural studies conducted under more environmentally relevant conditions, rather than the more confined and restricted laboratory conditions, may be justified (see Tan *et al*., 2020; Polverino *et al*., 2021).

Measuring uptake and effects on brain activity caused by APDs is also key to elucidating potential key areas of behavioural toxicity. Studies quantifying neurological responses alongside behavioural measures in fish are scarce (Scott & Sloman, 2004; Lürling & Scheffer, 2007; Gauthier & Vijayan, 2020). However, with the availability of several transgenic models capable of measuring brain activity *in vivo* (e.g elavl3:GCaMP6s; Winter *et al*., 2021), including in real time (Muto *et al*., 2013), and a range of established assays for quantifying behaviours relating to individual‐ and population‐level fitness in fish, the zebrafish has become one of the most popular fish models for investigating the effects of neuroactive pharmaceuticals on both individual fitness and population dynamics. In analyses of the impacts of APDs on behavioural measures in fish, the zebrafish features prominently (Gerlai, 2003; Sumanas & Lin, 2004; Kawahara *et al*., 2011; Khan *et al*., 2017; Audira *et al*., 2020) (Fig. 2). Of the APD‐relevant behavioural traits discussed here, some are integrated into existing apical endpoints in ERA, relating to individual survival, reproduction, and growth, while others, like predator avoidance, are currently absent from standard ecotoxicological studies.

### (2) Behavioural endpoints

#### (a) Sensory cues

The vulnerability of the sensory system to APD exposure is of key importance, given its role in so many behaviours in fish and therefore in overall fitness (Gerlach *et al*., 2007; Stengel, Wahby & Braunbeck, 2017). Only one study (Abreu *et al*., 2016) has examined the effects of APDs on chemosensory behaviour, and notably, this study did not expose fish to an environmentally relevant concentration or duration: risperidone was administered at 100 μg/l for just 150 s. It has been hypothesised that APDs could impair the sensitivity of the ocular system in fish, *via* accumulation in the fish eye and their role as inhibitors of aminergic activity, which typically modulate visual neurotransmission and responsiveness (Kirla *et al*., 2016; Bollmann, 2019). The paucity of sensory data however, makes it difficult to determine the mechanisms underlying neurotoxicity (e.g. potential retinotoxicity, ototoxicity, olfatoxicity, etc.) when observing behavioural phenotypes as a whole‐body response. Distinguishing between specific types of toxicity allows for strategic mechanism‐driven behavioural testing, as opposed to more generalised measures of ‘behaviour’. Additional behavioural endpoints (sensory taxis) and methods, including histological and molecular analysis, as well as perhaps more advanced techniques like eye tracking, will be necessary to distinguish explicitly sensory toxicity from other neurotoxic effects (Huber‐Reggi, Mueller & Neuhauss, 2012; Fitzgerald *et al*., 2021).

#### (b) Locomotion

Movement is a widely used endpoint in studies of behavioural ecotoxicology (Fig. 2; Boehmler *et al*., 2007). There is a close interplay between the dopaminergic (suppression) and serotonergic (stimulation) systems, which together modulate swimming speed, frequency, and movement patterns in fish (Little & Finger, 1990; Brustein *et al*., 2003; Thirumalai & Cline, 2008; Robinson, 2009; Montgomery *et al*., 2018). Exposure of zebrafish to APDs has been shown to disrupt neurotransmission in these systems and, accordingly, locomotion, although not at environmentally relevant concentrations or over chronic timeframes. An atypical APD, clozapine, has been shown to inhibit larval locomotion in zebrafish following differing exposure durations [116 h at 6.4 mg/l and 60 min at 4.1 mg/l, concentrations approximately 50–80‐fold greater than the maximum MEC found in fresh water (Boehmler *et al*., 2007; Gundlach *et al*., 2022)]. This effect was linked to the dopaminergic target D4R, with wider disruption of gene expression involved in monoaminergic function in the CNS. Risperidone (another atypical APD) has also been shown to affect locomotor activity (spontaneous larval swimming, following a 24‐h dosing period at 2.1 mg/l), but only transiently, despite a concentration over 500 times greater than the maximum MEC found in freshwater (Prieto *et al*., 2012). Acute exposure to haloperidol (from 5 min up to 2 h), a typical APD associated with movement disorders and extrapyramidal symptoms (EPSs), has been shown to induce hyper‐ or hypo‐locomotor activity, erratic or uncoordinated swimming, and aberrant positioning in both larval and adult zebrafish, with significant changes at concentrations as low as 2.7 μM [this still represents a concentration 10,000 times greater than the maximum MEC found in freshwater (Giacomini *et al*., 2006; Seibt *et al*., 2010; Irons *et al*., 2013; Magno *et al*., 2015)]. Giacomini *et al*. (2006) found that fluphenazine induced hypolocomotion at concentrations of 0.98 μM and above (this compound has not yet been detected in surface water). Chlorpromazine has also been shown to induce hypoactivity both at the early coiling stage in zebrafish embryos (at 39.2 μM, ~300,000 times the freshwater maximum MEC) and in free‐swimming larval zebrafish (exposed at 9.79 μM for 142 h, ~75,000 times the freshwater maximum MEC; Selderslaghs *et al*., 2013). Non‐target APD side effects such as convulsions are also likely contributors to potential disruption of locomotor circuitry and behaviour and require further research. Many of the effect concentrations reported, as illustrated above, are thousands‐fold greater than freshwater MECs. Nevertheless, behavioural studies examining the effects of APDs on more complex social behaviours (including predator avoidance and competition for resources, including food and mates) are lacking and these may be more sensitive than individual locomotor behaviour tests.

#### (c) Escape (fear) and avoidance (anxiety) behaviours with links to predator avoidance

Predator–prey relationships are directly related to animal fitness. Employing a repertoire of evasive and escape (Fig. 2) behaviours minimises risk to prey animals, thereby increasing their likelihood of survival in fluctuating environments (McEwen & Wingfield, 2003; Brown, 2003; Evans *et al*., 2019). Disruption of neurotransmission by APD compounds can modify anxiety responses at multiple levels (such as inaptitude or latency). For example, in cichlid fish, antagonism of serotonin receptor 5‐HT2A and its downstream effects on neurotransmission has been shown to increase startle probability and responsiveness to threat through changes in socially mediated startle‐escape plasticity in dominant fish (Whitaker *et al*., 2011). Furthermore, Jain *et al*. (2018), screening over 1000 APIs, found those affecting the serotonergic or dopaminergic system to be most likely to impair startle responses. Short‐term, relatively low‐level APD exposures (including at environmentally relevant concentrations) have been found to change critical anxiety and fear behavioural responses to stressful situations in exposed zebrafish and their offspring, making these fish less adept at responding to threat (discussed further below). In zebrafish, exposure to APDs, including clozapine exposure at 20–70 μg/l (below its freshwater maximum MEC of 78.3 μg/l; Table 1) for 72 h (Viana *et al*., 2020) and risperidone (at a concentration of 0.0003 μg/l and well below the freshwater maximum MEC of 3.68 μg/l) during the first 5 days of embryo development (Kalichak *et al*., 2019), have been found to reduce anxiety (as measured by a reduction in cortisol levels) and impair risk perception. The resulting adult zebrafish spend more time at the top of the tank (a higher risk position) than in the relatively safe zone at the bottom of the tank. Similarly, acute (15‐min) exposure to aripiprazole, including at an environmentally relevant concentration (5.56 ng/l, slightly below the freshwater maximum MEC of 8.3 ng/l), has been shown to dampen anti‐predator behaviours in adult zebrafish through suppression of cortisol production/release, albeit this did not occur in a concentration‐dependent manner (Barcellos *et al*., 2016, 2020). These consistent findings here, at (and below) environmentally realistic exposures, indicate the sensitivity and robustness of such behavioural endpoints for determining APD toxicity.

#### (d) Sociality (reproduction)

Sociality, the behavioural propensity to form groups, plays a crucial role in populations of social animals, including fish, by supporting the structure of social hierarchies, foraging and mating opportunities, reproductive success, and predator avoidance (Taborsky & Wong, 2017). Successful reproduction in vertebrates requires a well‐timed sequence of complex events and behaviours (Fig. 2). In fish, as in mammals, monoamines – specifically serotonin (stimulatory) and dopamine (inhibitory) – are simultaneously involved in the neuroendocrine regulation of gonadotropin‐releasing hormone (GnRH) (Lethimonier *et al*., 2004; Prasad *et al*., 2015b), which mediates the hypothalamo–pituitary–gonadal (HPG) axis, controlling the development and function of the reproductive system (Zohar *et al*., 2010; Fontaine *et al*., 2013; Kalarani, Vinodha & Moses, 2021). Little has been documented on the effects of APDs on reproduction in fish, let alone on reproductive behaviours such as courtship and mate selection. A single study on fathead minnows found no effect of the dopamine antagonist haloperidol (exposed for 21 days at 0.05 mg/l, 500× the freshwater maximum MEC) on reproductive output (Villeneuve *et al*., 2010a). Further studies of APDs on fish reproduction, including effects on mate selection and competition are warranted. More generally, other changes to sociality, with both enhancement and inhibition, have been reported for a number of apds: aripiprazole enhanced sociality in Mexican tetra (*Astyanax mexicanus*) albeit at 1 μM, which is over 50,000× the freshwater maximum MEC (Iwashita & Yoshizawa, 2021 ). Sociality in common carp has been shown to be altered by risperidone, and olanzapine for exposures at environmentally relevant concentrations or lower, specifically as low as 3.0 μg/l and 0.1 μg/l for these compounds, respectively (Chang *et al*., 2024).

#### (e) Social status, animal conflict and aggression

social hierarchies, territoriality and access to resources are mediated by aggression (Gurney & Nisbet, 1979; Popova, 2006; Fig. 2). Serotonin is known to reduce aggression and subordinate‐ranking fish commonly demonstrate elevated serontonin levels (Nelson & Chiavegatto, 2001; Filby *et al*., 2010; Lillesaar, 2011; Dahlbom *et al*., 2012). Studies on serotonin selective reuptake inhibitors (SSRIs) have mostly demonstrated reduced aggression in fish (Dzieweczynski & Hebert, 2012; Kohlert *et al*., 2012; Theodoridi, Tsalafouta & Pavlidis, 2017; Kellner *et al*., 2017). Dominant, naturally more aggressive fish are not only found to have lower expression of 5‐HTRs and serotonin activity, but they also often have increased dopaminergic activity, suggesting a system of competing monoaminergic neurotransmission, however studies have not reached a consensus regarding its effects on aggression (Elofsson *et al*., 2000; Winberg & Nilsson, 1992). Experimental effects of neuroactive pharmaceuticals on fish can include uniform changes in sociality and aggression (Kreke & Dietrich, 2008), as well as an increase in the social status and fitness of subordinate individuals (Whitaker *et al*., 2011). A study on the fathead minnow reported that chronic exposure to haloperidol, at 0.05 mg/l a concentration 500× the maximum freshwater MEC, increased dominance and success in competing for a breeding site (Villeneuve *et al*., 2010b). Chlorpromazine, the precursor to haloperidol, and with a similar MoA, has been shown to reduce aggression, and increase defensive behaviours, in chronically exposed blue acaras (*Aequidens pulcher*; Munro, 1986). Interestingly, however, chlorpromazine had no effect in medaka (Tsubokawa *et al*., 2009), nor haloperidol in zebrafish (Gutiérrez *et al*., 2019), albeit using acute rather than chronic exposures. None of these studies was undertaken at environmentally relevant concentrations and a more consistent approach will be required to understand the effects of APDs and other neuroactive compounds on fish sociality.

#### (f) Foraging

Foraging behaviour in fish (sporadic swimming interspersed with targeted prey capture) is regulated by the neuroendocrine system and is likely to be affected by pharmaceuticals, including APDs targeting monoaminergic systems (Carrillo & McHenry, 2016), particularly serotonin receptors (5‐HT1AR, 5‐HT1BR, 5‐HT2CR, 5‐HT4R) (Bacqué‐Cazenave *et al*., 2020). In humans, atypical APDs in particular are known to result in severe weight gain, as part of their induction of metabolic syndrome (Roerig, Steffen & Mitchell, 2011). The effects of APDs on foraging and feeding behaviour in fish have not yet been assessed, despite their potential to impair locomotion, speed, accuracy, motivation and metabolism. Gain in body mass and other metabolic effects, however, in response to atypical APD treatments have been noted in several studies on fish (De Alvarenga *et al*., 2016; Khanal, Patil & Unger, 2020; Xin *et al*., 2021; Chang *et al*., 2022). By contrast, studies on antidepressants indicate that increased serotonin (following inhibition of serotonin re‐uptake) reduces appetite and foraging in freshwater fish (Stanley *et al*., 2007; Gaworecki & Klaine, 2008; Hedgespeth, Nilsson & Berglund, 2014). Food intake and foraging ecology (within trophic cascades) are directly linked to animal fitness and have wider consequences for aquatic communities, making these associated behavioural endpoints important for ERA.

## IV. FUTURE PERSPECTIVES AND CHALLENGES FOR FISH BEHAVIOURAL ECOTOXICOLOGY

Both short‐term (acute) and long‐term (chronic) exposure of fish to neuroactive drugs, including APDs, has been shown to elicit quantifiable behavioural shifts at key life stages, from embryo hatching to adult fish mating. Current approaches in ERA are likely only to capture indirectly many aspects of behavioural outcomes, although not all, and not the nuances of specific traits as discussed above.

The current battery of regulatory fish tests includes partial (OECD 203, 210, 212, 215, 229, 236) and full life‐cycle assays (OECD 234, 240), which are standardised to address the effect of toxicants on one or multiple fitness‐relevant endpoints. While the specific data requirements of various global ERAs may vary, the OECD testing guidelines represent a good selection of standardised tests that could be adapted to incorporate behavioural endpoints, to some degree, to capture better the effects of neuroactive pharmaceuticals in ERA.

**Fig. 3:**
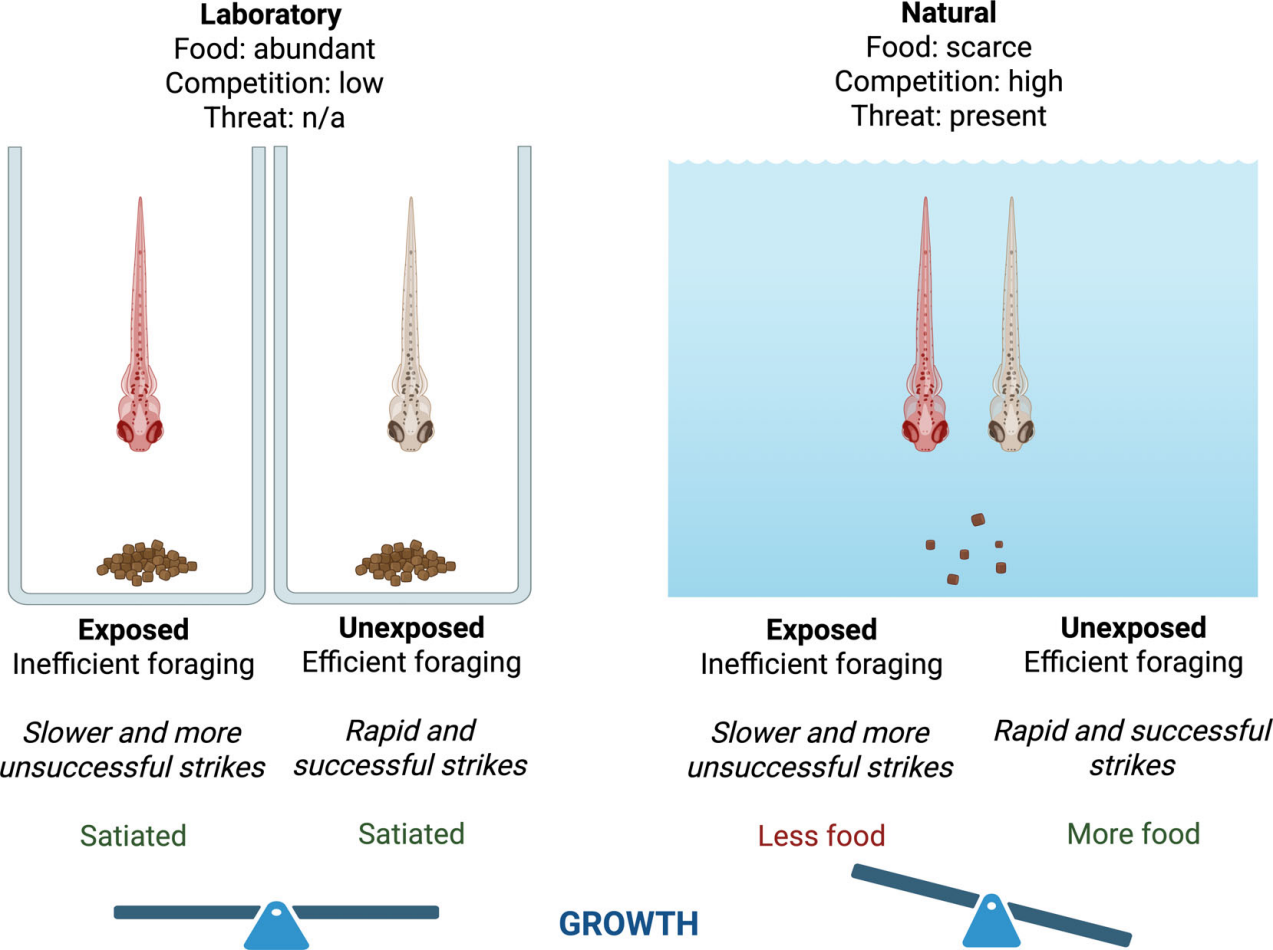
Consequences of the aggregation of feeding behaviour into a growth endpoint in environmental risk assessment (ERA), highlighting differences between laboratory and natural environments.

The assessment sensitivity detailed in the guidance documents, aimed at identifying markers of toxicity and relating these endpoints to potential population‐level effects, is compound dependent. Apical endpoints such as hatching, length, and abnormalities/mortality – often assessed within OECD test guidelines (e.g. OECD 210, 212, 215) – are related to general development and growth, thus indirectly capturing elements of sensory reception, locomotion and/or feeding at specific life stages. These endpoints are assessed due to their direct relevance to population survival. However, the specific mechanisms behind the endpoints – especially those that may indicate an APD‐driven behavioural change – are not captured. Increasing behavioural resolution could provide insights into areas of sub‐lethal toxicity that are otherwise aggregated into general endpoints. For example, decreased foraging efficiency may not be recognised or considered relevant within the food‐rich environment of a standardised assay, but could be more apparent in a food‐limited, competitive ‘real world’ scenario (Fig. 3). Growth as an endpoint in a laboratory study where food is not limiting, does not account for this. Furthermore, as for other neuroactive compounds, APDs may manifest toxicity only at or after a specific life stage (e.g. pre‐hatch embryos are often less susceptible to compound uptake), which may not be captured by these relatively short assays.

Assays such as OECD 229 (Fish Short‐Term Reproduction Assay) and OECD 234 (Fish Sexual Development Test) aim directly to capture changes in maturation, sexual development (differentiation) and consequently fitness (reproduction). These assays were developed to target endocrine‐disrupting compounds, and include a battery of specific tests including gonadal histopathology, vitellogenin quantification and assessment of secondary sex characteristics. Although gross morphological changes related to these biological effects would almost undoubtably affect behaviour, they are unlikely to occur at concentrations known to be environmentally relevant for APDs and their MoA, suggesting that reproductive behaviours would be more likely to be affected. However, current guidelines do not offer insight into specific behavioural strategies (mate choice and courtship) in their assessment of neuroactive compounds, only providing definitive endpoints (e.g. spawning), which can obscure more subtle underlying changes. Measuring the responses of fish following confined pairing of male and female fish in testing chambers, devoid of natural stressors, does not provide information on reproductive strategies such as breeding group size, courtship, competition, and social hierarchies. As a result, such assays are likely to be relatively insensitive to APD‐induced behavioural shifts. Moreover, recognising that exposure is likely to occur over a long period of time, the Fish Full Life Cycle test (OPPTS 850.1500) assesses the effects of chronic exposure on a number of standardised apical endpoints – on both the F0 adults and F1 offspring. However, the same caveats described above again apply, in addition to the added burden of a longer study duration, without necessarily increasing insight into toxicity of neuroactive compounds. Furthermore, some behavioural endpoints, such as predator avoidance, are not assessed by these tests even though they intuitively are related to survival.

High‐quality mechanistic endpoints relating to the specific MoA of APDs will be important to investigating their ecological relevance (in terms of survivability, development and reproduction) and to minimise uncertainty around their effects. Even if implemented only on an *ad hoc* basis specifically for neuroactive substances (rather than routinely, as was introduced for endocrine disrupting substances), such studies could provide valuable insights into potential risks. However, they are currently seen as too costly in terms of time and resources, and also require the development of standardised protocols for the reliable measurement of MoA‐relevant endpoints in different species and environments (i.e. with high predictive power). Furthermore, there is a need for greater clarification around thresholds that constitute an environmental concern.

### (1) Next generation of ERA for APDs and neuro‐pharmacology

Current standardised guidelines provide a basic template for more refined studies, including behavioural endpoints, to capture the potential risks of APDs and other neuroactive compounds (Bertram *et al*., 2025). To develop these existing assays, the application of modern tracking systems such as TRex and the use of artificial intelligence (AI) offer the ability to automate capture of fish responses. These technologies can analyse free‐swimming behaviour and identify and quantify key inter‐individual and individual–environment interactions (Walter & Couzin, 2021; Bertram *et al*., 2022).

Further work is required to assess behavioural scenarios, of increasing complexity, that are relevant to MoA‐related endpoints, to enable a wider perspective on the potential environmental effects of APD micropollutants (Bownik & Wlodkowic, 2021). Analysis of these effects will undoubtedly be supported by advances in AI methods to facilitate optimal data extraction, organisation, modelling and pattern detection (Hartung, 2023). Additionally, linking neurological responses at the level of the brain and CNS, particularly those associated with sensory systems and higher‐level behavioural functioning (e.g. cognition), will be crucial for a better understanding of the potential implications of neurotoxic pollutants on animal fitness (Jacquin *et al*., 2020). It should also be recognised that mixtures are naturally prevalent in the environment, and that pharmaceutical effects are likely to be complex, acting more widely on a range of targets, which may require a variety of assays to evaluate (Cleuvers, 2003; Pomati *et al*., 2007; Tornio *et al*., 2019). Few studies have investigated mixtures of APDs (although see David *et al*., 2018), despite their pervasiveness in the environment: APDs are the most prevalently detected (13%) group of contaminants in European surface waters (Busch *et al*., 2016; Ogungbemi *et al*., 2021). Interestingly, a 1:1 mixture of the APDs sulpiride and clozapine weakened their effects on monoaminergic expression in zebrafish, and in some cases, led to a reduction in transcription responses when compared to their individual treatment (Zhang *et al*., 2022).

There is increasing evidence that genetic polymorphisms within the genes coding for serotonin and dopamine receptors contribute to inter‐individual variation in drug action in humans. Other relevant polymorphisms include those in G‐proteins, which initiate signal transduction, as well as transport proteins and degrading enzymes, which potentiate and attenuate signalling within the serotonin and dopamine systems (Bondy & Zill, 2004). Our review clearly illustrates there are major limitations in our understanding of the effects of chronic APD exposure in fish, particularly in the roles of genotypic variation and phenotypic plasticity in physiological and behavioural responses to APDs. There is an urgent need for quantitative, mechanistically driven and ecologically relevant studies, incorporating full‐life cycle, transgenerational exposure, and depuration/recovery assessments (see Table 2) to evaluate the most potent APDs and support confident regulatory decision‐making (Meredith‐Williams *et al*., 2012, Hird *et al*., 2016).

**Table 2:** Outstanding questions to be addressed by academics and industry regulators.

Industry
What are the perceived barriers to considering sublethal behavioural endpoints in ERA?
Where are the greatest opportunities for including behavioural endpoints in ERA?
When and how should the effects of chemical and pharmaceutical mixtures be taken into consideration?
Should significant differences in behaviour endpoints be weighted equally to apical endpoints?
Academics
How do potential manifestations of drug effects, e.g. sensory system deficits, influence behaviour?
How should we standardise studies? What stimuli should we employ, if any?
How should variation and plasticity in behavioural response be accounted for in ERA (genetic background, age/sex, level of conditioning/domestication, adequate level of replication)?
Can alteration in behavioural phenotypes following sublethal chemical/pharmaceutical exposure be linked to adverse outcomes for individual organisms [impaired survival, growth and/or reproduction in the laboratory and in the field]?
Will individual adverse outcomes translate to population‐level adverse outcomes (in the environment) i.e. what are the consequences for natural selection?

### (2) Standardisation and domestication

Standardisation and consistent reporting of behavioural studies, including specifying the genetic background and level of domestication of test organisms, is of paramount importance. Genetic strains and lines need to be specified and maintained between behavioural assays to ensure robust interpretation of results (Pan, Chatterjee & Gerlai, 2012; Vignet *et al*., 2013; Audira *et al*., 2020). For studies employing zebrafish, environmental relevance is offered by wild‐type strains with high genetic diversity and low levels of domestication, while reproducibility is offered by more inbred, domesticated strains (Brown *et al*., 2012). However, the genetics of some of the longest‐established and well‐used zebrafish strains [e.g. Wild Indian Kolkata (WIK) strain derived from a single‐pair mating of wild‐caught fish in 1997], have historically been poorly managed, leading to genetic drift and inconsistency between sub‐strains (Crim & Lawrence, 2021). The highly controlled, constrained, and unstimulating rearing environments for model freshwater fish have also been found to cause shrinkage in multiple brain regions, further limiting their behavioural repertoires (Price, 1999; Salvanes & Braithwaite, 2005), as well as ‘emotional’ welfare (Stevens, Reed & Hawkins, 2021; Lee, Paull & Tyler, 2022).

Domestication can also reduce behavioural plasticity, the capacity to express a variety of behavioural phenotypes from the same genotype, thereby limiting genotypic resilience for optimised population performance in heterogeneous environments (Saaristo *et al*., 2018). Wild populations, which experience a wider range of environmental conditions, are more likely to retain a greater level of behavioural plasticity compared to domestic laboratory strains (Sih & Bell, 2008; Thoré, Brendonck & Pinceel, 2021). In fact, when comparing wild and captive‐bred strains of zebrafish, Coe *et al*. (2009) found that the genetic diversity of the wild populations was richer and more variable. However, creating a perfect natural setting in which to study pharmaceutically induced behaviour is challenging and typically outside the scope of most ecotoxicological studies (Huntingford, 2004). Recording behavioural endpoints in response to pharmaceutical exposure in natural field environments is even more challenging. Therefore, it is important to recognise that a laboratory study performed on a single behavioural axis can provide a pragmatic solution for measuring the effects of APDs on specific behaviours (e.g. reproductive behaviours), despite overlooking other potential behavioural axes and influences, which may be present in the environment (Sih, Bell & Johnson, 2004; Sih & Bell, 2008, Sih, Ferrari & Harris 2011; Sih, 2013; Bertram *et al*., 2022).

### (3) Individual behaviour to population‐level responses

Other key factors that must be considered for the use of behaviours in assessing the risks of APDs (and other neuroactive chemicals) include quantifying: (*i*) variation in behaviour within and among individuals to evaluate behavioural plasticity; (*ii*) changes in behavioural responses to gradients of external stimuli (reaction norms); and (*iii*) links between behavioural endpoints and significant impacts on individual and population fitness in the wild (Sumpter, 2014). Erosion of inter‐ and intra‐individual behavioural diversity in fish could be indicative of an overwhelming effect of pharmaceutical pollution. Although this is a key area of interest, no evidence is yet available linking effects on individual behaviours to wider ecological implications. Research on better‐characterised psychoactive compounds, such as fluoxetine, has shown that exposure can constrain behavioural variation and population resilience in fish (Polverino *et al*., 2021; Aich *et al*., 2024). Ultimately, such effects could have wider implications for the stability and adaptive capacity of populations under anthropogenic stress.

**Fig. 4:**
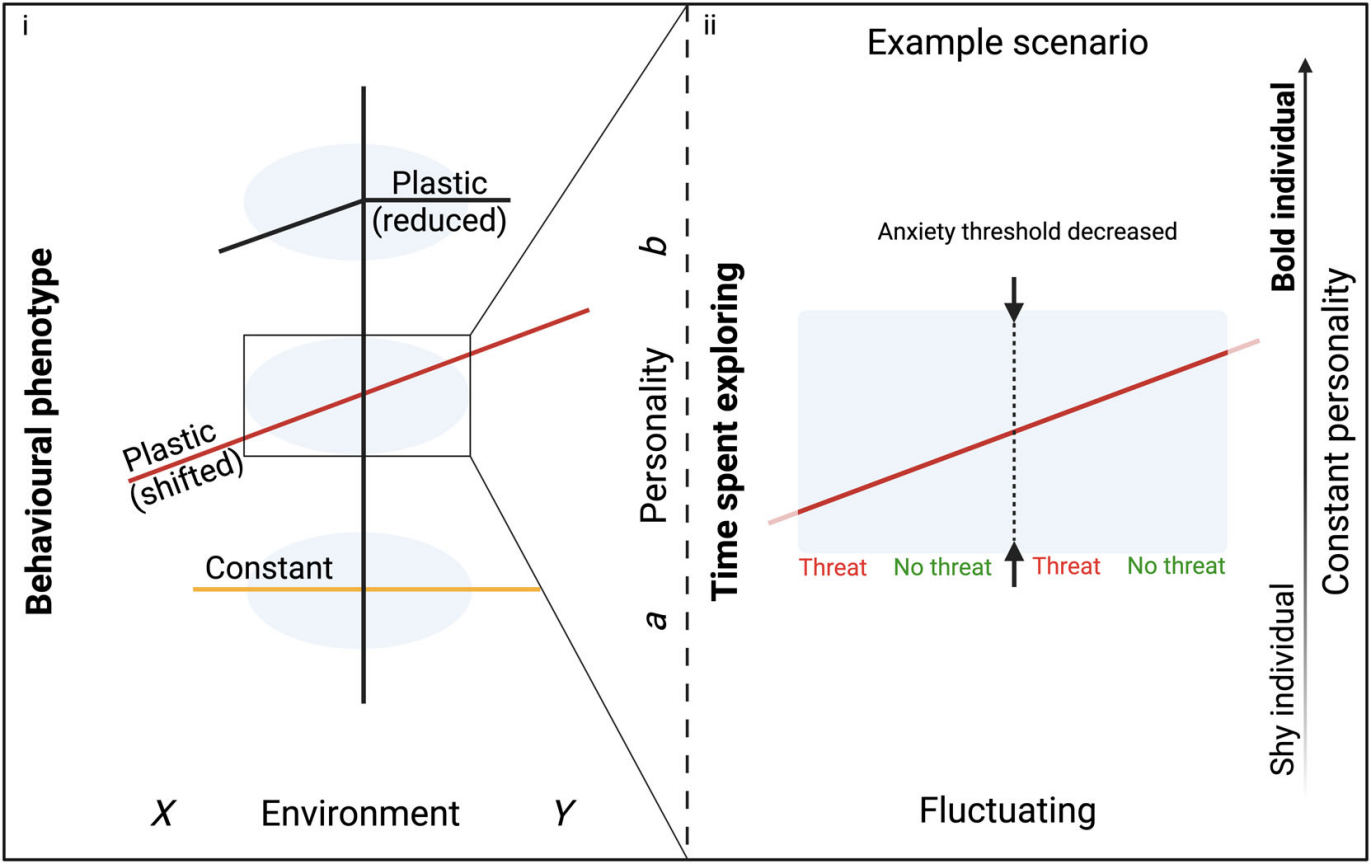
Understanding behavioural plasticity and personality. (A) Illustration of conceptual reaction norms, demonstrating differences in response to an environmental change from *X* to *Y* constrained within personality type *a* or *b*. The range of behavioural responses can be visualised through quantitative graphical presentations as reaction norms, representing behaviour as a range of responses within an environmental context, e.g. predator threat or habitat disturbance (Dingemanse *et al*., 2010). The use of reaction norms and clustering can be applied to high‐quality behavioural data in a powerful framework to provide an integrated view of both plasticity (the behavioural range of the population) and personality (average behaviours of individuals) within an ecological and evolutionary context (Sarkar, 1999). (B) Example scenario magnified from A detailing how a plastic trait [exploration under negative (Threat, e.g. predator threat) and neutral (No threat, i.e. perceived safety) environmental stimuli] can be shifted in response to pharmaceutical exposure, within the boundaries of its personality type, whereby a shy personality type constrains the individual from spending more time exploring.

Early development and formative experiences are often implicated in functionally altering inter‐ and intra‐individual variation (plasticity) in behaviour. This variation results from changes in neuro‐expression in the brain to accommodate the perceived future needs of the organism. Such processes have been observed in various fish species including cod (*Gadus morhua*) and Atlantic salmon (*Salmo salar*) (Salvanes, Moberg & Braithwaite, 2007; Salvanes *et al*., 2013; Groothuis & Taborsky, 2015). It is therefore important to acknowledge determinants of behavioural differences (Table 3), including genetic and environmental (experiential) factors, when comparing studies (Voelkl *et al*., 2020). A more nuanced understanding of the complexity of these phenotypic variables (see Table 3) will enhance experimental rigor and improve consistency across studies. Important in this regard is the recognition of biological variation and responses associated with genetically hardwired (innate) and experience‐based (learned) behaviours. It is also essential to recognise that the rate of environmental change may erode or exceed the boundaries of a behaviour and its associated plasticity (i.e. move it beyond the reaction norm), causing the organism to become mismatched with its environment. This can result in an inability to match cues accurately with responses, leading to an evolutionary trap and ultimately, population decline (Badyaev, 2005; Snell‐Rood, 2013; Herborn *et al*., 2014; Arnett & Kinnison, 2016). The erosion of inter‐ and intra‐individual differences also could indicate overwhelming effects of environmental agents such as pharmaceutical contamination with neuroactive compounds (Aich *et al*., 2024). Understanding the broader ecological consequences and evolutionary trajectories of fish following exposure to APDs therefore requires assessment of behavioural phenotypic plasticity, genotypic resilience and population stability (van Der Goot *et al*., 2021), as well as a wider appreciation of correlated behaviours (behavioural syndromes) and individually consistent ‘personality’ types (Fig. 4) (Réale *et al*., 2007; Dingemanse *et al*., 2010; Dzieweczynski *et al*., 2016). Behavioural plasticity exists in the constraining presence of personality (Fig. 4), and these elements are complementary for maintaining population variation (Sih *et al*., 2004; Réale *et al*., 2007; Dingemanse *et al*., 2010). Bell, Hankison & Laskowski (2009) associated up to 35% of inter‐population phenotypic variation with animal personality, although personality is individually consistent regardless of environmental context (Réale *et al*., 2007). As illustrated in Fig. 4, boldness and associated behavioural syndromes, including the willingness to explore *versus* reluctance and shyness, tend to persist, despite the potential adaptive advantage conferred by the ability to switch personality type (Dammhahn & Almeling, 2012; Langerhans *et al*., 2004). To understand better how behaviour is affected by exposure to APDs, assessing the levels of ‘background’ variation in behaviours will provide greater ecological validity to the interpretation of any observed effects (Dzieweczynski *et al*., 2016; van Der Goot *et al*., 2021).

**Table 3:** Determinants of behavioural differences for a number of key behaviours with potential for use for environmental risk assessment. For each behaviour, its function, wider ecological consequences, its genetic or learned basis, and its plasticity/individual variation is provided. Understanding complex phenotypic variables in this way will enable greater experimental understanding and consistent research practice.

Behaviour	Individual level function	Ecological/population consequences	Innate/learned	Plastic/personality effect
Spontaneous coiling	Dechorionation	Initiation of movement	Innate	No
Startle response	Escape (rapid defence against threat)	Predator avoidance (increased survival)	Innate	Underlying plastic circuitry (synaptic plasticity)
Sleep phenotypes	Circadian homeostasis (memory, learning, energy conservation)	Equilibrated (attentive and healthy) population within space and time	Innate	No
(Photo/chemo)taxis	Cue‐based navigation in a fluctuating environment	Accurate approach or avoidance of key positive (e.g. food) and negative (e.g. dangerous) stimuli	Innate	No
Locomotion	Activity for resource procurement and risk minimisation	Dispersal and migration; imbalanced population dynamics can result if mating/resource procurement is reduced or predation risk increased	Innate	Yes
Thigmotaxis	Safety	Secondary‐level predator avoidance (population equilibrium within the ecosystem)	Innate	No
Foraging	Feeding success	Food web cascades	Innate and learned (e.g. to modify foraging in different environments, patch choice, prey detection/capture and assessment of nutrient content)	Yes: foraging flexibility and opportunity to forage
Sociality	Position in social hierarchy (reproductive success)	Social connectedness and community (cross‐boundary effects)	Innate sociality and social learning	Yes: including forming social hierarchies
Courtship	Reproductive success/output	Population maintenance	Largely innate	Yes: within courtship and preferences
Anxiety and fear	Escape and avoidance	Complex predator avoidance (balanced population dynamics)	Innate and learned (e.g. predator recognition and risk assessment)	Yes: varying responses to stress, and predator threat
Learning: novelty and habituation	Appropriate environmental reactivity	Equilibrated (attentive and active) population within space and time	Innate motivation to react to novelty followed by learned habituation response as it is encountered further	Yes: type and speed of reactions

### (4) A note on animal use ethics

For any chemical testing, it is necessary to consider animal welfare and the use of appropriate substitute methods, e.g. *in vitro*/*in silico* tools (Coors *et al*., 2023). Behavioural ecotoxicity assays are typically undertaken at lower concentrations than those that induce overt toxicity, and are generally non‐invasive. Each animal also can potentially be employed for multiple behavioural assays or repeated measures within chronic assays, maximising data acquisition per organism and thus reducing the overall number of animals used. Such assays are likely less distressing than more invasive methods, thus plausibly also minimising overall animal suffering (Thoré *et al*., 2021).

## V. CONCLUSIONS

(1) Antipsychotic drugs (APDs) are underrepresented in environmental risk assessments (ERAs) yet are considered a hazardous class of neuroactive compounds. These pharmaceuticals have the potential to cause significant harmful effects on fish by altering neural processes, affecting behaviour and, ultimately individual and/or population fitness. However, most current standard ecotoxicological tests employed in ERA do not include behavioural effects in a standardised or quantitative manner. While some standard ecotoxicology tests do incorporate some behavioural aspects qualitatively (e.g. OECD 203), they are not included within ERA.
(2) It is also the case that effects of APDs have yet to be explored adequately in ‘non‐standard’ research studies. Limited existing evidence indicates that sublethal effects on individual fish in the laboratory can occur for environmentally relevant exposures, indicating possible impacts on wild populations. The behavioural effects observed likely also depend on the different underlying modes of action of different classes of APDs.
(3) Behavioural outcomes are likely to be somewhat species and situation specific, depending on trophic position and habitat (e.g. availability of shelter, foraging and spawning areas). Identifying sensitive periods and ecologically relevant (specific and robust) behaviours with important fitness consequences (e.g. impaired predator avoidance correlates with a higher probability of predation and lower fitness) will be crucial steps to understand better how the nature and magnitude of responses may affect fitness.
(4) Tailoring ERA for groups of neuroactive drugs, such as APDs, will require the standardisation and refinement of behavioural ecotoxicology. This will necessitate a deeper understanding of how to interpret effects on key behavioural endpoints in the context of population viability, together with the integration of relevant CNS‐related molecular and physiological markers. Increasingly robust behavioural assays, performed in the laboratory and incorporating a range of measured behaviours along with appropriate stimuli where necessary, should be considered for inclusion in ERA of neuroactive substances.

## AUTHOR CONTRIBUTIONS

The manuscript was written by G. W.‐B., A. R. B. and C. R. T., with subsequent input and editing contributions from all authors. All authors approved the final version. The opinions expressed herein are those of the authors only and do not necessarily reflect the opinion of the institutions to which the authors are affiliated or the opinion of all PREMIER partners.

## Supporting information


**Table S1.** Toxicity interactions found following antipsychotic drug exposure in fish.
