# Supplementary material for: Critical insights into the potential risks of antipsychotic drugs to fish, including through effects on behaviour
Source: Biol Rev Camb Philos Soc. 2025 May 12;100(5):1994–2019. doi: 10.1111/brv.70031 (PMC12407060; doi:10.1111/brv.70031)
Supplement: Supplementary file 1 — Table S1. Toxicity interactions found following antipsychotic drug exposure in fish. [file BRV-100-1994-s001.docx]

**Table S1.** Toxicity interactions found following antipsychotic drug (APD) exposure in fish. Literature searched and selected on electronic databases, supplemented with sources found from references and additional sources. Searches were specified for fish species, availability of at least one detailed measure of a toxicity (endpoint) and APD exposure (all APDs with available information are listed here: ARP, aripiprazole; CLZ, clozapine; CPM, chlorpromazine; CYA, cyamemazine; FLU, fluphenazine; HAL, haloperidol; OLZ, olanzapine; RIS, risperidone; SUL, sulpride; TFL, trifluoperazine; ZIP, ziprasidone). dpf, days post-fertilisation; hpf, hours post-fertilisation; n.a, not available. All concentrations under investigation are listed. The effect endpoint and effect description are summarised for clarity. # indicates that sex-specific effects were reported.

| **Drug** | **Fish species (life-stage and sex)** | **Acute or chronic exposure** | **Concentration studied (μg/l conversion if molarity is reported)** | **Effect endpoint** | **Effect description** | **Reference** |
| --- | --- | --- | --- | --- | --- | --- |
| ARP | (*Astyanax mexicanus*) Mexican tetra strains (adult; sex not stated) | Acute (overnight ~16 h) | 1 μM (448 μg/l) | Locomotion and sociality | Reduced overall swimming distance and speed. The cave-dwelling strain also showed reduced turn bias and increased social interactions | Iwashita & Yoshizawa (2021) |
|  | (*Danio rerio*) Zebrafish (adult; mixed sex) | Acute (15 min) | 0.556, 5.56, and 556 ng/l | Antipredator behaviour | Impairs antipredator response | Barcellos *et al*. (2020) |
|  | Mexican tetra (adult; sex not stated) | Acute (overnight ~16 h) | 1–5 μM (448–2242 μg/l) | Sleep, locomotion and attraction (approach) behaviour | Reduced attraction and hyperactivity (swimming distance) and increased sleep duration | Yoshizawa *et al*. (2018) |
|  | Zebrafish (adult; mixed sex, 50:50 male and female) | Acute (15 min) | 0.0556, 0.556, 5.556, 55.6 and 556 ng/l | Stress responses (cortisol levels) | Blunted stress response | Barcellos *et al*. (2016) |
|  | Zebrafish (larvae; indeterminate sex) | Acute (1 h incubation + time to measure behaviour) | 1.25, 2.5, 5, 10, 20, 40, 80 μM  (560–35,871 μg/l) | Visual (light)- and acoustic-stimulated locomotion | Dose-dependent change in ‘motion index’ (motor activity) | Bruni *et al*. (2016) |
|  | Zebrafish (Larvae; indeterminate sex) | Semi-chronic (3 days) | 10 - 30 μM (4484–13,452 μg/l) | Rest/wake behaviour | Inappropriate hyperactivity in the dark | Rihel *et al*. (2010) |
| **CLZ** | Zebrafish (larvae; indeterminate sex) | Semi-chronic (116 h) | 0.2, 0.4, 0.8, 1.6, 3.2 and 6.4 mg/l | Locomotion, development and genomics | Decrease in average swim distance and velocity. Body length reduction at the highest concentration. Relevant target genes upregulated from middle concentration | Gundlach *et al*. (2022) |
|  | (*Gobiocypris rarus*) Chinese rare minnows (2 months old juveniles; # mixed sex) | Chronic (90 days) | 0.1, 1, and 10 μg/l | Lipotoxicity | Increased mass and induced gut microbiome dysbiosis, hepatic lipid droplet accumulation and dyslipidemia | Xin *et al*. (2021) |
|  | Zebrafish (adult; # mixed sex) | Acute (72 h) | 20–70 µg/l | Locomotion and tank position | Dose-dependent effect on swimming behaviour and tank position; inappropriate time spent at the top of the tank | Viana *et al*. (2020) |
|  | Mexican tetra (adult; sex not stated) | Acute (overnight ~16 h) | 0.1–12.5 μM  (32.682–4,085 μg/l) | Sleep, locomotion and attraction behaviour (approach to vibration) | Reduced attraction and hyperactivity (swimming distance) and increased sleep duration | Yoshizawa *et al*. (2018) |
|  | (*Pimephales promelas*) Fathead minnow (adult) and (*Ictalurus punctatus*) channel catfish (juvenile). Sex not stated | Semi-chronic: 28 days or 7 days | 50 μg/l | Bioconcentration potential (following depuration) | Persistent accumulation in body tissues, especially the brain | Nallani *et al*. (2016) |
|  | Zebrafish: larvae (5dpf; indeterminate sex) | Acute (12 min) | 1, 3 and 10 μM  (326.8–3,268 μg/l) | Intestinal motility | Significantly reduced frequency of gut contractions | De Alvarenga *et al*. (2016) |
|  | Zebrafish: larvae (5dpf; indeterminate sex) | Acute (1 h incubation + time to measure behaviour) | 1.25, 2.5, 5, 10, 20, 40, 80 μM (408.5–26,146 μg/l) | Visual (light)- and acoustic-stimulated locomotion | Dose-dependent change in ‘motion index’ (motor activity) | Bruni *et al*. (2016) |
|  | Fathead minnow: embryo to larval (indeterminate sex) | Chronic (48 hpf – 28 dpf) | 6.25,12.5, 25, 50, 100 μg/l | Survival and growth | Decreased survival, increased growth and induced abnormalities | Overturf *et al*. (2012) |
|  | Zebrafish (embryo; indeterminate sex) | Semi-chronic (0 hpf – 6 dpf) | 1 µg/l – 10 mg/l | Fish Embryo Acute Toxicity Endpoints | Coagulation, eye defects, pericardial oedema, absence of circulation and malformations | Akande *et al*. (2010) |
|  | Zebrafish: larval to 7 dpf  (indeterminate sex) | Acute (60 min) | 12.5 μM (4,085 μg/l) | Locomotion | Inhibition of locomotion (hypoactivity) | Boehmler *et al*. (2007) |
| **HAL** | (*Clarias gariepinus*) African sharptooth catfish (juvenile; sex not stated) | 15 days followed by 5 days after drug withdrawn | 0.12, 0.24, 0.48 mg/l | Genotoxicity, neural activity and depuration | Sustained increase in micronuclei, abated effects on neural activity following drug withdrawal | Chiejina *et al*. (2023) |
|  | Freshwater African catfish (juvenile; sex not stated) | 15 days followed by 5 days after drug withdrawn | 0.12, 0.24, 0.48 mg/l | Locomotion, haematological and biochemical parameters | Reduction in swimming rate and increased hyperactivity. Improvement in hematological and biochemical parameters following withdrawal | Chiejina *et al*. (2022) |
|  | Zebrafish (juvenile; sex-matched) | Acute (30 min acclimatisation + recording time) | 1,10, 50 μM (375.9–18,795 μg/l) | Locomotor and aggression | No significant changes | Gutiérrez *et al*. (2019) |
|  | Zebrafish (larval; indeterminate sex) | Repeated chronic 0–6 dpf, late acute (1 h) and early transient (24 h) | 1, 10, and 100 μg/l | Locomotion and response to light | Decreased swimming activity (by 25–40% in repeated exposure and by 31% with early transient exposure) and hypoactivity in light | Huang *et al*. (2019) |
|  | Zebrafish: larval (6 dpf; indeterminate sex) | Semi-chronic (5 hpf – 5 dpf) | 0.5 or 1.5 μM (188–564 μg/l) | Response to light | Inappropriate hyperactivity in the dark and hypoactivity in the light | Oliveri & Levin (2019) |
|  | (*Salmo trutta*) Brown trout (age n.a; sex not stated) | Chronic (1–18 months) | Field exposure | Bioaccumulation | Found in fish tissues (particularly kidneys and liver) | Grabicová *et al*. (2017) |
|  | Zebrafish (adult; sex not stated) | Acute (90 min) | 0.625, 1.25, 2.5, 5, 10 μM  (235–3,759 μg/l) | Locomotion and anxiety-like behavioural responses (novel tank test) | Endpoints were variably affected depending on the concentration | Tran *et al*. (2016) |
|  | Zebrafish (larvae; indeterminate sex) | 1 h incubation + time to measure behaviour | 1.25, 2.5, 5, 10, 20, 40, 80 μM  (470–30,072 μg/l) | Visual (light)- and acoustic-stimulated locomotion | Reduced activity at high concentrations (20–80 μM). Lower concentrations (2.5–10 μM), altered the behavioural profile of movement but not the average activity | Bruni *et al*. (2016) |
|  | Zebrafish (adult; mixed sex) | Acute (300 s) | 1,2, 4, 8 mg/l | Swimming activity and response to light | Inappropriately decreased time spent in the dark and increased motor activity | Magno *et al*. (2015) |
|  | Zebrafish (larval; indeterminate sex) | Acute (10–20 min acclimatisation followed by 70 – 260 min) | 0.2–50 µM (75–18,795 μg/l) | Response to light | Decreased activity and blunted response to light, e.g. hyperactive in the dark | Irons *et al*. (2013) |
|  | Zebrafish (larvae; indeterminate sex) | Acute (30 min acclimation followed by 80 min) | 1, 2.5, 5 μM (376–1,880 μg/l) | Locomotion | HAL was not found to affect activity levels. When HAL was co-administered with Pentylenetetrazole it exacerbated changes (increase) in activity | Ellis & Soanes (2012) |
|  | Zebrafish (adult; mixed sex) | Acute (30 min) | 9 μM (3,383 μg/l) | Locomotion | Postural unbalance and erratic swimming patterns/general coordination issues | Seibt *et al*. (2010) |
|  | Fathead minnow (adult 6-months old; sex-differentiated) | Chronic (21 days) | 0.02, 0.2, 2, 20 μg/l | Reproduction | No statistically significant treatment-related difference in fecundity, fertility, and body condition (including gonads). Increase in gonadotropin-releasing hormone (GnRH) transcripts in males | Villeneuve *et al*. (2010*a*) |
|  | Fathead minnow (adult 6-months old) and zebrafish (adult 5-months old; mixed sex) | Acute (96 h) | 50 μg/l | Reproduction and aggression (nesting behaviour) | In fathead minnows HAL increased aggression phenotype. Altered genetic expression in the female ovary of both fathead minnows and zebrafish (though not found to be a robust marker) | Villeneuve *et al*., (2010*b*) |
|  | Zebrafish (larvae; indeterminate sex) | Acute (2 h) | 9 μM (3,383 μg/l) | Motor response | Movement deficit (bouts of erratic swimming) | Giacomini *et al*. (2006) |
| **RIS** | (*Cyprinus carpio*) Common carp (age n.a; sex-differentiated) | Chronic (60 days) | 0.03 and 3 μg/l | Locomotion (distance, speed, acceleration) and social interaction | Altered (decreased) swim parameters and inhibited sociality | Chang *et al*. (2024) |
|  | Seven native Portuguese (estuarine) species (late juveniles/ young adults); sex not stated | Field derived | Not detected in grab sampling | Bioaccumulation | High detection frequency in fish tissues (72, 65, 69%) respectively in brain, muscle and liver tissues, at concentrations between 0.1 and 0.6 ng/g | Duarte *et al*. (2023) |
|  | Zebrafish (larvae; indeterminate sex) | Acute (30 min or 24 h incubation + 4 h tracking) | 5 μM (2,052 μg/l) | Locomotion and light–dark test | Hypoactivity after longer exposure (24 h) | Banono *et al*. (2020) |
|  | Zebrafish (embryo and larvae; indeterminate sex) | Semi-chronic (0 hpf – 5 dpf) | 0.0003, 0.003, 0.03 μg/l | Antipredator behaviour, persistence, survival, hatching, and heart rates | Persistently impaired antipredatory behaviour (hyperactivity). Also reduced survival, hatching, and heart rates | Kalichak *et al*. (2019) |
|  | Zebrafish (juvenile; sex-matched) | Acute (30 min acclimatisation + recording time) | 3, 6, 12 μM (1,231–4,926 μg/l) | Locomotion and aggression | Not significant | Gutiérrez *et al*. (2019) |
|  | Zebrafish (larvae; indeterminate sex) | Repeated chronic 0–6 dpf, late acute (1 h) and early transient (24 h) | 1, 10, and 100 μg/l | Swimming activity and response to light | No effect | Huang *et al*. (2019) |
|  | Mexican tetra (adult; sex not stated) | Acute (overnight ~16 h) | 1 – 5 μM (410–2,052 μg/l) | Sleep, locomotion and attraction (approach) behaviour | Reduced attraction and hyperactivity (swimming distance) and increased sleep duration | Yoshizawa *et al*. (2018) |
|  | Zebrafish (larvae; indeterminate sex) | Semi-chronic (0 hpf – 5 dpf) | 0.00034, 0.003, and 0.03 µg/l | Anxiety and mortality | Altered exploration (hyperactivity), as well as mortality | Kalichak *et al*. (2017) |
|  | Zebrafish (embryo–larvae; indeterminate sex) | Semi-chronic (0 hpf - 5dpf) | 0.00033, 0.0033, 0.033, 0.33, 3.3, 33 μg/l | Mortality, development and reproductive success | Impaired larval development (size), survival and heart rate | Kalichak *et al*. (2016) |
|  | Zebrafish (adult; mixed sex) | Acute (150 s) | 0.00034, 100, 170 μg/l | Chemosensory behaviour | Inappropriate attraction to RIS | Abreu *et al*. (2016) |
|  | Zebrafish (larvae; indeterminate sex) | 1 h incubation + time to measure behaviour | 2.5, 5, 10, 20, 40, 80, 160 μM (1,026–65,678 μg/l) | Visual (light)- and acoustic-stimulated locomotion | Dose-dependent change in ‘motion index’ (motor activity) | Bruni *et al*. (2016) |
|  | Zebrafish (adult; mixed sex) | Acute (15 min) | 0.00034, 85, 170, 340 and 680 μg/l | Stress | Decreased stress response | Idalencio *et al*. (2015) |
|  | Zebrafish (adult; mixed sex) | Acute (300 s) | 0.1, 0.25, 0.5 mg/l | Locomotion and response to light (anxiety) | No effect | Magno *et al*. (2015) |
|  | Zebrafish (larvae 4 or 6 dpf; indeterminate sex) | Acute (24h) | 5 μM (2.1 mg/l) | Locomotion | Transiently effected locomotion | Prieto *et al*. (2012) |
| **OLZ** | Common carp (age n.a; sex-differentiated #) | Chronic (60 days) | 0.1 and 10 μg/l | Locomotion (distance, speed, acceleration) and social interaction | Altered (decreased) swim parameters and inhibited sociality | Chang *et al*. (2024) |
|  | Common carp (age n.a; sex not stated) | Chronic (60 days) | 10, 100, and 250 μM (3,124–78,110 μg/l) | Lipotoxicity | Altered gut microbiota and relevant changes to lipid metabolism (mass gain, change to cholesterol and lipoprotein levels and lipid accumulation in the liver) | Chang *et al*. (2022) |
|  | Zebrafish: embryo–larvae (72 hpf) and adult (female) | Acute 24 h and semi-chronic (21 days) | 0.5–200 μM (156–62,488 μg/l) | Lipotoxicity | Lipid accumulation, weight gain and hyperphagia | Khanal *et al*. (2020) |
|  | Zebrafish (larvae; indeterminate sex) | 1 h incubation + time to measure behaviour | 2.5, 5, 10, 20, 40, 80, 160 μM (781–49,990 μg/l) | Visual (light)- and acoustic-stimulated locomotion | Dose-dependent change in ‘motion index’ (motor activity) | Bruni *et al*. (2016) |
|  | Zebrafish (adult; mixed sex) | Acute (15–30 min) | 100 μM (31,244 μg/l) | Locomotion and space use in a tank | Significant anxiolytic response | Seibt *et al*. (2010) |
|  | Zebrafish (larvae; indeterminate sex) | Acute (2 h) | 9 μM (2,812 μg/l) | Motor response | Minor significant effect on locomotion | Giacomini *et al*. (2006) |
| **SUL** | Zebrafish (adult; mixed sex) | Acute (20 min) | 10 & 100 μM (3,414–34,143 μg/l) | Swim pattern [changes to THC (tetrahydrocannabinol)-induced circling as a psychosis-like phenotype] | No effect on normal locomotion. When SUL was co-administered with THC, repetitive circling was specifically ameliorated, without influencing overall velocity | Dahlén *et al*. (2021) |
|  | Zebrafish (larvae; indeterminate sex) | Acute (1 h) co-administered with N-ethylpentylone (NEP) | 10 μM (3,414 μg/l) | Locomotion (free swim) and thigmotaxis | Offset changes in locomotion caused by NEP. No effect on thigmotaxis | Fan *et al*. (2021) |
|  | Zebrafish (adult; mixed sex) | Acute (1 h) | 5, 10, 20 mg/l | Memory (Y-maze side search alteration) | No effect | Cleal *et al*. (2020) |
|  | Zebrafish (adult; male) | Acute (1 h) | 10 μM (3,414 μg/l) | Locomotion and space use | No effect on wild-type behaviour. Changed (increased bottom dwelling) anxiety-like response to cocaine | Kacprzak *et al*. (2017) |
|  | Zebrafish (adult; sex not stated) | Acute (30 min before co-administration) | 50 mg/kg (50,000 μg/l) | Locomotion and anxiety | No effect | Da Silva *et al*. (2015) |
|  | Zebrafish (larvae; indeterminate sex) | Acute (30 min acclimation followed by 80 min) | 250–1000 μM (85,357–341,427 μg/l) | Locomotion | No effect on normal activity or co-administered Pentylenetetrazole-induced activity changes | Ellis & Soanes. (2012) |
|  | Zebrafish (adult; mixed sex) | Acute (15–30 min) | 250 μM (85,357 μg/l) | Locomotion and space use | No effect | Seibt *et al*. (2010) |
| **CPM** | Zebrafish (embryo; indeterminate sex) | Acute (30–150 min) | 1 & 10 μM (319–3,189 μg/l) | Photomotor response | Supressed normal photomotor response (at the highest concentration) | Gauthier & Vijayan (2019) |
|  | Zebrafish (eleutheroembryos and larvae; indeterminate sex) | Semi-chronic (2–144 hpf) | 4.89×10^−3^ to 3.92×10^−2^ mM (1,559–12,501 μg/l) | Developmental neurotoxicity (*via* early movement) | Hypoactivity (lower mean duration of tail coiling) and teratogenicity | Selderslaghs *et al*. (2013) |
|  | (*Oryzias latipes*) Medaka (adult; sex-differentiated #) | Acute (30 min post-injection) | 15 and 30 mg/kg | Shoaling behaviour (swimming and mirror approach) | Suppressed distance swam. No effect on mirror approach | Tsubokawa *et al*. (2009) |
|  | (*Carassius auratus*) Goldfish (adult; sex not stated) | Acute (30 min – 12 h), semi-chronic (3 days) & chronic 15–21 days | 0.001, 0.005, 0.01, 0.05 and 0.1 mg/l | Behaviour and oxidative stress | Abnormal erratic behaviour, and induced oxidative stress (decreased enzyme activity) | Li *et al*. (2008) |
|  | (*Andinoacara pulcher*) Blue acaras (maturing; female) | Chronic (32 days) | 0.25 – 2 mg/l (250–2000 µg/l) | Antagonistic behaviour | Reduced aggression, increased defensive behaviour, and fin resting (sedative action or fear-induced immobility) | Munro (1986) |
| **FLU** | Zebrafish (larvae 7 or 14 dpf; indeterminate sex) | Acute (40–45 h or 2 h) | 0.98 and  1.57 μM or 9 μM (0.4–0.64 or 3.65 mg/l) | Motor response | After 40–45 h exposure of 14 dpf fish, FLU decreased swim speed. After 2 h in FLU, 7 dpf moved slower and more erratically | Giacomini *et al*. (2006) |
| **TFL** | Zebrafish (juvenile; sex-matched) | Acute (30 min acclimatisation + recording time) | 0.01, 0.1, 1 μM (4.075–407.5 μg/l) | Locomotor and aggression | Not significant | Gutiérrez *et al*. (2019) |
| **CYA** | Japanese medaka (larvae; indeterminate sex) | Semi-chronic (hatch – 72 h) | 1.22, 12.2, 122, 1,222 μg/l | Locomotion and space use | Biphasic dose–response curve: increased locomotion  at low and intermediate concentrations, but hypoactivity (and thigmotaxis) only at high concentrations. | Chiffre *et al*. (2014) |
| **ZIP** | Zebrafish (juvenile; sex-matched) | Acute (30 min acclimatisation + recording time) | 0.1, 1, 10 μM (41–4,129 μg/l) | Locomotor and aggression | Not significant | Gutiérrez *et al*. (2019) |

**References**

Abreu, M. S., Giacomini, A. C., Gusso, D., Da Rosa, J. G. S., Koakoski, G., Kalichak, F., Idalêncio, R., Oliveira, T. A., Barcellos, H. H. A., Bonan, C. D. & Barcellos, L. J. G. (2016). Acute exposure to waterborne psychoactive drugs attract zebrafish. *Comparative Biochemistry and Physiology Part C: Toxicology & Pharmacology* *179*, 37–43. https://doi.org/10.1016/j.cbpc.2015.08.009

Akande, M. G., Orn, S. & Norrgren, L. (2010). Evaluation of the Toxic Effects of Clozapine in Zebra fish (*Danio rerio*) embryos with the Fish Embryo Toxicity Test. *International Journal of Biological & Pharmaceutical Research* **1**, 71–75.

Banono, N. S., Gawel, K., De Witte, L. & Esguerra, C. V. (2020). Zebrafish Larvae Carrying a Splice Variant Mutation in cacna1d: A New Model for Schizophrenia-Like Behaviours? *Molecular Neurobiology* *58*(2), 877–894. https://doi.org/10.1007/s12035-020-02160-5

Barcellos, H. H., Kalichak, F., Rosa, J. L. G., Oliveira, T. Y., Koakoski, G., Idalencio, R., De Abreu, M. S., Giacomini, A. C., Fagundes, M., Variani, C., Rossini, M., Piato, A. L. S. & Barcellos, L. J. G. (2016). Waterborne aripiprazole blunts the stress response in zebrafish. *Scientific Reports*, *6*(1). <https://doi.org/10.1038/srep37612>

Barcellos, H. H., Pompermaier, A., Mendonça-Soares, S., Maffi, V. C., Fernandes, M., Koakoski, G., Kirsten, K., Baldisserotto, B. & Barcellos, L. J. G. (2020). Aripiprazole prevents stress-induced anxiety and social impairment, but impairs antipredatory behavior in zebrafish. *Pharmacology, Biochemistry and Behavior* *189*, 172841. <https://doi.org/10.1016/j.pbb.2019.172841>

Boehmler, W., Carr, T. D., Thisse, C., Thisse, B., Canfield, V. A. & Levenson, R. W. (2007). D4 Dopamine receptor genes of zebrafish and effects of the antipsychotic clozapine on larval swimming behaviour. *Genes, Brain and Behavior* *6*(2), 155–166. <https://doi.org/10.1111/j.1601-183x.2006.00243.x>

Bruni, G., Rennekamp, A. J., Velenich, A., McCarroll, M., Gendelev, L., Fertsch, E., Taylor, J., Lakhani, P., Lensen, D., Evron, T., Lorello, P. J., Huang, X., Kolczewski, S., Carey, G., Caldarone, B. J., Prinssen, E., *et al*. (2016). Zebrafish behavioral profiling identifies multitarget antipsychotic-like compounds. *Nature Chemical Biology* *12*(7), 559–566. <https://doi.org/10.1038/nchembio.2097>

Chang, X., Shen, Y., Yun, L., Wang, X., Feng, J., Yang, G., Meng, X., Zhang, J. & Su, X. (2022). The antipsychotic drug olanzapine altered lipid metabolism in the common carp (Cyprinus carpio L.): Insight from the gut microbiota-SCFAs-liver axis. *The Science of the Total Environment* *856*, 159054. https://doi.org/10.1016/j.scitotenv.2022.159054

Chang, X., Shen, Y., Yang, M., Yun, L., Liu, Z., Feng, S., Yang, G., Meng, X. & Su, X. (2024). Antipsychotic drug-induced behavioral abnormalities in common carp: The potential involvement of the gut microbiota-brain axis. *Journal of Hazardous Materials* *472*, 134444. https://doi.org/10.1016/j.jhazmat.2024.134444

Chiejina, C. O., Anih, L. N., Okoye, C. O., Aguzie, I. O., Ali, D., Kumar, G. & Nwani, C. D. (2022). Haloperidol alters the behavioral, hematological and biochemical parameters of freshwater African catfish, Clarias gariepinus (Burchell 1822). *Comparative Biochemistry and Physiology Part C: Toxicology & Pharmacology*, *254*, 109292. <https://doi.org/10.1016/j.cbpc.2022.109292>

Chiejina, C. O., Ikeh, I. M., Enebe, F. A., Aguzie, I. O., Ajima, M. N. O., Ali, D., Kumar, G. & Nwani, C. D. (2023). Effects of haloperidol on peripheral erythrocytes and brain neurotransmitter levels of juvenile African Sharptooth Catfish Clarias gariepinus. *Journal of Aquatic Animal Health* *35*(4), 238–247. https://doi.org/10.1002/aah.10195

Chiffre, A., Clérandeau, C., Dwoinikoff, C., Bihanic, F. L., Budzinski, H., Geret, F. & Cachot, J. (2014). Psychotropic drugs in mixture alter swimming behaviour of Japanese medaka (Oryzias latipes) larvae above environmental concentrations. *Environmental Science and Pollution Research*, *23*(6), 4964–4977. https://doi.org/10.1007/s11356-014-3477-4

Cleal, M., Fontana, B. D., Ranson, D. C., McBride, S. D., Swinny, J. D., Redhead, E. S. & Parker, M. O. (2020). The Free-movement pattern Y-maze: A cross-species measure of working memory and executive function. *Behavior Research Methods*, *53*(2), 536–557. https://doi.org/10.3758/s13428-020-01452-x

Dahlén, A., Zarei, M., Melgoza, A., Wagle, M. & Guo, S. (2021). THC-induced behavioral stereotypy in zebrafish as a model of psychosis-like behavior. *Scientific Reports*, *11*(1). <https://doi.org/10.1038/s41598-021-95016-4>

Da Silva, R. B., Siebel, A. M. & Bonan, C. D. (2015). The role of purinergic and dopaminergic systems on MK-801-induced antidepressant effects in zebrafish. *Pharmacology Biochemistry and Behavior* *139*, 149–157. https://doi.org/10.1016/j.pbb.2015.05.005

De Alvarenga, K. a. F., Sacramento, E. K., Rosa, D. V., Souza, B. R., De Rezende, V. B. & Romano-Silva, M. A. (2016). Effects of antipsychotics on intestinal motility in zebrafish larvae. *Neurogastroenterology and Motility*, *29*(5). <https://doi.org/10.1111/nmo.13006>

Duarte, I. A., Reis-Santos, P., Fick, J., Cabral, H., Duarte, B. & Fonseca, V. F. (2023). Neuroactive pharmaceuticals in estuaries: Occurrence and tissue-specific bioaccumulation in multiple fish species. *Environmental Pollution* *316*, 120531. <https://doi.org/10.1016/j.envpol.2022.120531>

Ellis, L. D. & Soanes, K. H. (2012). A larval zebrafish model of bipolar disorder as a screening platform for neuro-therapeutics. *Behavioural Brain Research*, *233*(2), 450–457. <https://doi.org/10.1016/j.bbr.2012.05.043>

Fan, E., Xu, Z., Yan, J., Wang, F., Sun, S., Zhang, Y., Zheng, S., Wang, X. & Rao, Y. (2021). Acute exposure to N-Ethylpentylone induces developmental toxicity and dopaminergic receptor-regulated aberrances in zebrafish larvae. *Toxicology and Applied Pharmacology* *417*, 115477. <https://doi.org/10.1016/j.taap.2021.115477>

Gauthier, P. T. & Vijayan, M. M. (2019). A rapid zebrafish embryo behavioral biosensor that is capable of detecting environmental β-blockers. *Environmental Pollution* *250*, 493–502. <https://doi.org/10.1016/j.envpol.2019.03.053>

Giacomini, N. J., Rose, B., Kobayashi, K. & Guo, S. (2006). Antipsychotics produce locomotor impairment in larval zebrafish. *Neurotoxicology and Teratology*, *28*(2), 245–250. https://doi.org/10.1016/j.ntt.2006.01.013

Grabicová, K., Grabic, R., Fedorova, G., Fick, J., Cerveny, D., Kolarova, J., Turek, J., Zlabek, V. & Randak, T. (2017). Bioaccumulation of psychoactive pharmaceuticals in fish in an effluent dominated stream. *Water Research* *124*, 654–662. <https://doi.org/10.1016/j.watres.2017.08.018>

Gundlach, M. J., Di Paolo, C., Chen, Q., Majewski, K., Haigis, A., Werner, I. & Hollert, H. (2022). Clozapine modulation of zebrafish swimming behavior and gene expression as a case study to investigate effects of atypical drugs on aquatic organisms. *Science of the Total Environment*, *815*, 152621. <https://doi.org/10.1016/j.scitotenv.2021.152621>

Gutiérrez, H. C., Vacca, I., Schoenmacker, G., Cleal, M., Tochwin, A., O’Connor, B., Young, A. M., Vasquez, A. A., Winter, M. J., Parker, M. O. & Norton, W. H. (2019). Screening for drugs to reduce zebrafish aggression identifies caffeine and sildenafil. *European Neuropsychopharmacology*, *30*, 17–29. https://doi.org/10.1016/j.euroneuro.2019.10.005

Huang, I. J., Sirotkin, H. I. & McElroy, A. E. (2019). Varying the exposure period and duration of neuroactive pharmaceuticals and their metabolites modulates effects on the visual motor response in zebrafish (Danio rerio) larvae. *Neurotoxicology and Teratology*, *72*, 39–48. <https://doi.org/10.1016/j.ntt.2019.01.006>

Idalencio, R., Kalichak, F., Rosa, J. L. G., De Oliveira, T. F., Koakoski, G., Gusso, D., De Abreu, M. S., Giacomini, A. C., De Alcantara Barcellos, H. H., Piato, A. L. S. & Barcellos, L. J. G. (2015). Waterborne risperidone decreases stress response in zebrafish. *PLOS ONE* *10*(10), e0140800. <https://doi.org/10.1371/journal.pone.0140800>

Irons, T., Kelly, P. E., Hunter, D. L., MacPhail, R. C. & Padilla, S. (2013). Acute administration of dopaminergic drugs has differential effects on locomotion in larval zebrafish. *Pharmacology, Biochemistry and Behavior* *103*(4), 792–813. <https://doi.org/10.1016/j.pbb.2012.12.010>

Iwashita, M. & Yoshizawa, M. (2021). Social-like responses are inducible in asocial Mexican cavefish despite the exhibition of strong repetitive behavior. *eLife* *10*. <https://doi.org/10.7554/elife.72463>

Kacprzak, V., Patel, N. A., Riley, E., Yu, L., Yeh, J. J. & Zhdanova, I. V. (2017). Dopaminergic control of anxiety in young and aged zebrafish. *Pharmacology Biochemistry and Behavior* *157*, 1–8. https://doi.org/10.1016/j.pbb.2017.01.005

Kalichak, F., De Alcantara Barcellos, H. H., Idalencio, R., Koakoski, G., Soares, S. M., Pompermaier, A., Rossini, M. & Barcellos, L. J. G. (2019). Persistent and transgenerational effects of risperidone in zebrafish. *Environmental Science and Pollution Research* *26*(25), 26293–26303. <https://doi.org/10.1007/s11356-019-05890-9>

Kalichak, F., Idalêncio, R., Da Rosa, J. G. S., De Alcântara Barcellos, H. H., Fagundes, M., Piato, Â. & Barcellos, L. J. G. (2017). Psychotropic in the environment: risperidone residues affect the behavior of fish larvae. *Scientific Reports* *7*(1). https://doi.org/10.1038/s41598-017-14575-7

Kalichak, F., Idalêncio, R., Da Rosa, J. G. S., Oliveira, T. A., Koakoski, G., Gusso, D., De Abreu, M. S., Giacomini, A. C., Barcellos, H. H. A., Fagundes, M., Piato, Â. & Barcellos, L. J. G. (2016). Waterborne psychoactive drugs impair the initial development of Zebrafish. *Environmental Toxicology and Pharmacology* *41*, 89–94. https://doi.org/10.1016/j.etap.2015.11.014

Khanal, P., Patil, B. M. & Unger, B. S. (2020). Zebrafish shares common metabolic pathways with mammalian olanzapine-induced obesity. *Future Journal of Pharmaceutical Sciences* *6*(1). <https://doi.org/10.1186/s43094-020-00049-7>

Li, T., Zhou, Q., Zhang, N. & Luo, Y. (2008). Toxic effects of chlorpromazine on Carassius auratus and its oxidative stress. *Journal of Environmental Science and Health, Part B, Pesticides, Food Contaminants, and Agricultural Wastes* *43*(8), 638–643. https://doi.org/10.1080/03601230802352674

Magno, L. D. P., Fontes, A., Gonçalves, B. & Gouveia, A. (2015). Pharmacological study of the light/dark preference test in zebrafish (Danio rerio): Waterborne administration. *Pharmacology, Biochemistry and Behavior* *135*, 169–176. <https://doi.org/10.1016/j.pbb.2015.05.014>

Munro, A. D. (1986). The effects of apomorphine, d-amphetamine and chlorpromazine on the aggressiveness of isolated Aequidens pulcher (Teleostei, Cichlidae). *Psychopharmacology* *88*(1), 124–128. <https://doi.org/10.1007/bf00310527>

Nallani, G. C., Edziyie, R., Paulos, P. M., Venables, B. J., Constantine, L. A. & Huggett, D. B. (2016). Bioconcentration of two basic pharmaceuticals, verapamil and clozapine, in fish. *Environmental Toxicology and Chemistry* *35*(3), 593–603. <https://doi.org/10.1002/etc.3244>

Oliveri, A. N. & Levin, E. D. (2019). Dopamine D1 and D2 receptor antagonism during development alters later behavior in zebrafish. *Behavioural Brain Research*, *356*, 250–256. https://doi.org/10.1016/j.bbr.2018.08.028

Overturf, M. D., Overturf, C. L., Baxter, D. W., Hala, D., Constantine, L. A., Venables, B. J. & Huggett, D. B. (2012). Early Life-Stage Toxicity of Eight Pharmaceuticals to the Fathead Minnow, Pimephales promelas. *Archives of Environmental Contamination and Toxicology*, *62*(3), 455–464. <https://doi.org/10.1007/s00244-011-9723-6>

Rihel, J., Prober, D. A., Arvanites, A. C., Lam, K. L., Zimmerman, S. C., Jang, S., Haggarty, S. J., Kokel, D., Rubin, L. L., Peterson, R. T. & Schier, A. F. (2010). Zebrafish behavioral profiling links drugs to biological targets and rest/wake regulation. *Science* *327*(5963), 348–351. https://doi.org/10.1126/science.1183090

Seibt, K. J., Da Luz Oliveira, R., Zimmermann, F. F., Capiotti, K. M., Bogo, M. R., Ghisleni, G. & Bonan, C. D. (2010). Antipsychotic drugs prevent the motor hyperactivity induced by psychotomimetic MK-801 in zebrafish (Danio rerio). *Behavioural Brain Research* *214*(2), 417–422. <https://doi.org/10.1016/j.bbr.2010.06.014>

Selderslaghs, I. W., Hooyberghs, J., Blust, R. & Witters, H. E. (2013). Assessment of the developmental neurotoxicity of compounds by measuring locomotor activity in zebrafish embryos and larvae. *Neurotoxicology and Teratology* *37*, 44–56. https://doi.org/10.1016/j.ntt.2013.01.003

Tran, S., Facciol, A. & Gerlai, R. (2016). Alcohol-induced behavioral changes in zebrafish: The role of dopamine D2-like receptors. *Psychopharmacology*, *233*(11), 2119–2128. https://doi.org/10.1007/s00213-016-4264-3

Tsubokawa, T., Saito, K., Kawano, H., Kawamura, K., Shinozuka, K. & Watanabe, S. (2009). Pharmacological effects on mirror approaching behavior and neurochemical aspects of the telencephalon in the fish, medaka (Oryzias latipes). *Social Neuroscience* *4*(3), 276–286. <https://doi.org/10.1080/17470910802625215>

Viana, J., Wildman, N., Hannon, E., Farbos, A., Neill, P., Moore, K., Van Aerle, R., Paull, G., Santos, E. M. & Mill, J. (2020). Clozapine-induced transcriptional changes in the zebrafish brain. *Npj Schizophrenia* *6*(1). <https://doi.org/10.1038/s41537-019-0092-x>

Villeneuve, D. L., Garcia-Reyero, N., Martinović, D., Mueller, N. D., Cavallin, J. E., Durhan, E. J., Makynen, E. A., Jensen, K. M., Kahl, M. D., Blake, L. S., Perkins, E. J. & Ankley, G. T. (2010a). I. Effects of a dopamine receptor antagonist on fathead minnow, Pimephales promelas, reproduction. *Ecotoxicology and Environmental Safety*, *73*(4), 472–477. https://doi.org/10.1016/j.ecoenv.2009.09.007

Villeneuve, D. L., Garcia-Reyero, N., Martinović, D., Mueller, N. D., Cavallin, J. E., Durhan, E. J., Makynen, E. A., Jensen, K. M., Kahl, M. D., Blake, L. S., Perkins, E. J. & Ankley, G. T. (2010b). II: Effects of a dopamine receptor antagonist on fathead minnow dominance behavior and ovarian gene expression in the fathead minnow and zebrafish. *Ecotoxicology and Environmental Safety*, *73*(4), 478–485. https://doi.org/10.1016/j.ecoenv.2009.09.018

Xin, J., Yan, S., Hong, X., Zhang, H. & Zha, J. (2021). Environmentally relevant concentrations of clozapine induced lipotoxicity and gut microbiota dysbiosis in Chinese rare minnow (Gobiocypris rarus). *Environmental Pollution*, *286*, 117298. <https://doi.org/10.1016/j.envpol.2021.117298>

Yoshizawa, M., Settle, A., Hermosura, M. C., Tuttle, L. J., Cetraro, N., Passow, C. N. & McGaugh, S. E. (2018). The evolution of a series of behavioral traits is associated with autism-risk genes in cavefish. *BMC Evolutionary Biology*, *18*(1). <https://doi.org/10.1186/s12862-018-1199-9>
